# OsNRT1.1B‐OsCNGC14/16‐Ca^2+^‐OsNLP3 Pathway: Phosphorylation‐Mediated Maintenance of Nitrogen Homeostasis

**DOI:** 10.1002/advs.202507919

**Published:** 2025-09-03

**Authors:** Xiaohan Wang, Yongqiang Liu, Weiwei Li, Xiaojun Ma, Wei Wang, Zhimin Jiang, Yiqin Wang, Legong Li, Bin Hu, Chengcai Chu

**Affiliations:** ^1^ Institute of Genetics and Developmental Biology Chinese Academy of Sciences Beijing 100101 China; ^2^ Institute of Crop Sciences Chinese Academy of Agricultural Sciences Beijing 100081 China; ^3^ Guangdong Basic Research Center of Excellence for Precise Breeding of Future Crops and Guangdong Laboratory for Lingnan Modern Agriculture College of Agriculture South China Agricultural University Guangzhou Guangdong 510642 China; ^4^ Key Laboratory for Enhancing Resource Use Efficiency of Crops in South China Ministry of Agriculture and Rural Affairs College of Agriculture South China Agricultural University Guangzhou Guangdong 510642 China; ^5^ College of Life Sciences Capital Normal University Beijing 100048 China

**Keywords:** Ca^2^⁺ signaling, nitrate Signaling, nitrogen homeostasis, phosphorylation, rice

## Abstract

Nitrate, a crucial nutrient and signaling molecule, is extensively studied across plants. While the NRT1.1‐NLP‐centered pathway dominates nitrate signaling in *Arabidopsis* and rice, however, whether there is functional interaction or co‐regulation between the primary nitrate response (PNR) and long‐term nitrogen utilization remains unclear. Here, a novel nitrate signaling pathway is identified in rice that works alongside the established ubiquitination‐mediated OsNRT1.1B‐OsSPX4‐OsNLP3 cascade. It is demonstrated that OsCNGC14, OsCNGC16, and OsNRT1.1B form a plasma membrane‐localized complex in root tips, mediating nitrate‐triggered Ca^2^⁺ influx. The absence of either OsCNGC14 or OsCNGC16 abolished Ca^2^⁺ signaling and suppressed PNR. The OsNRT1.1B‐OsCNGC14/16 complex activates Ca^2^⁺‐dependent phosphorylation of OsNLP3 at Ser193, which accelerates its nuclear translocation and transcriptional activation of nitrate‐responsive genes. This phosphorylation enhances both short‐term PNR and long‐term nitrogenutilization. This findings reveal a dual regulatory network in rice: the Ca^2^⁺‐OsNLP3 pathway rapidly amplifies nitrate signals, while the ubiquitination‐mediated OsSPX4 degradation ensures sustained nitrogen homeostasis.

## Graphical abstract

OsNRT1.1B forms a membrane complex with OsCNGC14/16 that mediates nitrate‐triggered calcium influx. This calcium signal phosphorylates OsNLP3 at Ser193, accelerating its nuclear translocation and activating nitrogen‐responsive genes. The calcium‐dependent pathway complements the established ubiquitination‐mediated pathway, dynamically regulating nitrogen homeostasis and enhancing nitrogen utilization efficiency in rice.

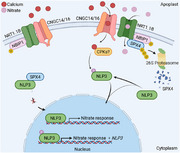

## 1 Introduction

Nitrogen (N) availability profoundly impacts plant growth, development, and agricultural productivity,^[^ ^1^, ^2^, ^3^ ^]^ and consequently shapes global primary production.^[^ ^4^ ^]^ However, excessive dependence on synthetic fertilizers poses economic and environmental challenges.^[^ ^5^, ^6^, ^7^ ^]^ Nitrate, a primary N source for plants, serves both as a nutritional element and a signaling molecule, regulating transcriptional and physiological adaptations.^[^ ^3^, ^8^, ^9^ ^]^

The initiation of nitrate signaling involves specialized transceptors: AtNRT1.1 in *Arabidopsis* and OsNRT1.1B in rice.^[^ ^10^, ^11^ ^]^ Upon activation, these transceptors stimulate master transcriptional regulators—AtNLP7 in *Arabidopsis* and OsNLP3 in rice—by triggering their nuclear translocation to regulate the expression of nitrate‐responsive genes.^[^ ^12^, ^13^ ^]^ However, the downstream cytoplasmic signaling mechanisms exhibit striking divergence between these two model plants.^[^ ^14^ ^]^ In *Arabidopsis*, nitrate perception by AtNRT1.1 elicits cytosolic Ca^2^⁺ fluxes that activate Ca^2^⁺‐sensor protein kinases (CPKs). These CPKs phosphorylate AtNLP7, stabilizing its nuclear localization and sustaining transcriptional activation of nitrate‐responsive genes.^[^ ^15^, ^16^ ^]^ In contrast, rice utilizes a distinct ubiquitination‐mediated pathway: OsNRT1.1B perceives nitrate and subsequently recruits the E3 ligase OsNBIP1. This interaction triggers the degradation of the transcriptional repressor OsSPX4, thereby releasing OsNLP3 from cytoplasmic sequestration. As a result, OsNLP3 translocates to the nucleus, where it activates nitrate‐responsive genes.^[^ ^13^ ^]^

Notably, AtNLP7‐mediated signaling cascade in *Arabidopsis* generates rapid transcriptional responses to N availability, characterized by distinct primary (minutes) and secondary (hours) phases that ultimately promote plant growth in response to nitrate.^[^ ^6^, ^17^, ^18^ ^]^ Despite this progress, the integration of these rapid signals with long‐term N utilization remains poorly understood. Therefore, establishing a unified nitrate signaling network that integrates rapid external nitrate sensing with adaptive responses for long‐term N utilization is critical for improving crop nitrogen use efficiency (NUE). Such a model would not only elucidate the mechanistic basis of primary nitrate response (PNR) but also provide actionable insights for sustainable agriculture.

Here, we address this critical gap by identifying a Ca^2^⁺‐dependent nitrate signaling pathway in rice that functions synergistically with the canonical ubiquitination cascade. We demonstrate that OsCNGC14 and OsCNGC16, two cyclic nucleotide‐gated channels, interact with OsNRT1.1B to transduce nitrate signal into intracellular Ca^2^⁺ fluxes. This Ca^2^⁺ signaling leads to the phosphorylation of OsNLP3 and amplifies its transcriptional activity. This dual regulatory mechanism enables rice to dynamically balance immediate nitrate sensing with sustained N utilization.

## 2 Results

### 2.1 A Calcium‐Dependent Pathway Complements the Ubiquitination Cascade in Rice Nitrate Signaling

Nitrate, an essential N source for plants, is well‐documented for its role in activating the PNR signaling pathway.^[^ ^10^, ^11^, ^15^, ^17^, ^19^, ^20^ ^]^ While numerous genes involved in PNR, including transporters, N assimilation enzymes, and transcription factors, have been implicated in regulating NUE,^[^ ^11^, ^21^, ^22^, ^23^, ^24^, ^25^, ^26^, ^27^, ^28^ ^]^ the hierarchical organization of PNR‐controlled nitrate signaling in rice and its role in long‐term N utilization remain poorly understood. To address this, we performed time‐course RT‐qPCR analyses of nitrate‐responsive genes, revealing a biphasic induction pattern: an initial transcriptional peak at 1 h (PNR phase; Figure S1, Supporting Information) followed by a secondary peak at 4–5 h for *OsNRT2.1*, *OsNIA*, and *OsNIR* (except for *OsGS1.1*) (Figure S1A–C, Supporting Information). To dissect these two regulatory phases, we analyzed nitrate induction in the *osnrt1.1b* and *osnbip1‐1* mutants. Strikingly, both induction peaks were abolished in *osnrt1.1b* (Figure S1, Supporting Information), whereas *osnbip1‐1* mutant retained the secondary peak at levels comparable to wild‐type (Figure S1, Supporting Information). This indicates that *osnbip1* specifically disrupts PNR without impairing the secondary nitrate response. Notably, this PNR‐independent secondary activation in rice diverges from the canonical NLP‐mediated cascade in *Arabidopsis*,^[^ ^29^ ^]^ suggesting the existence of a parallel regulatory pathway operating alongside the OsNRT1.1B‐OsSPX4‐OsNLP3 axis.

**Figure 1:**
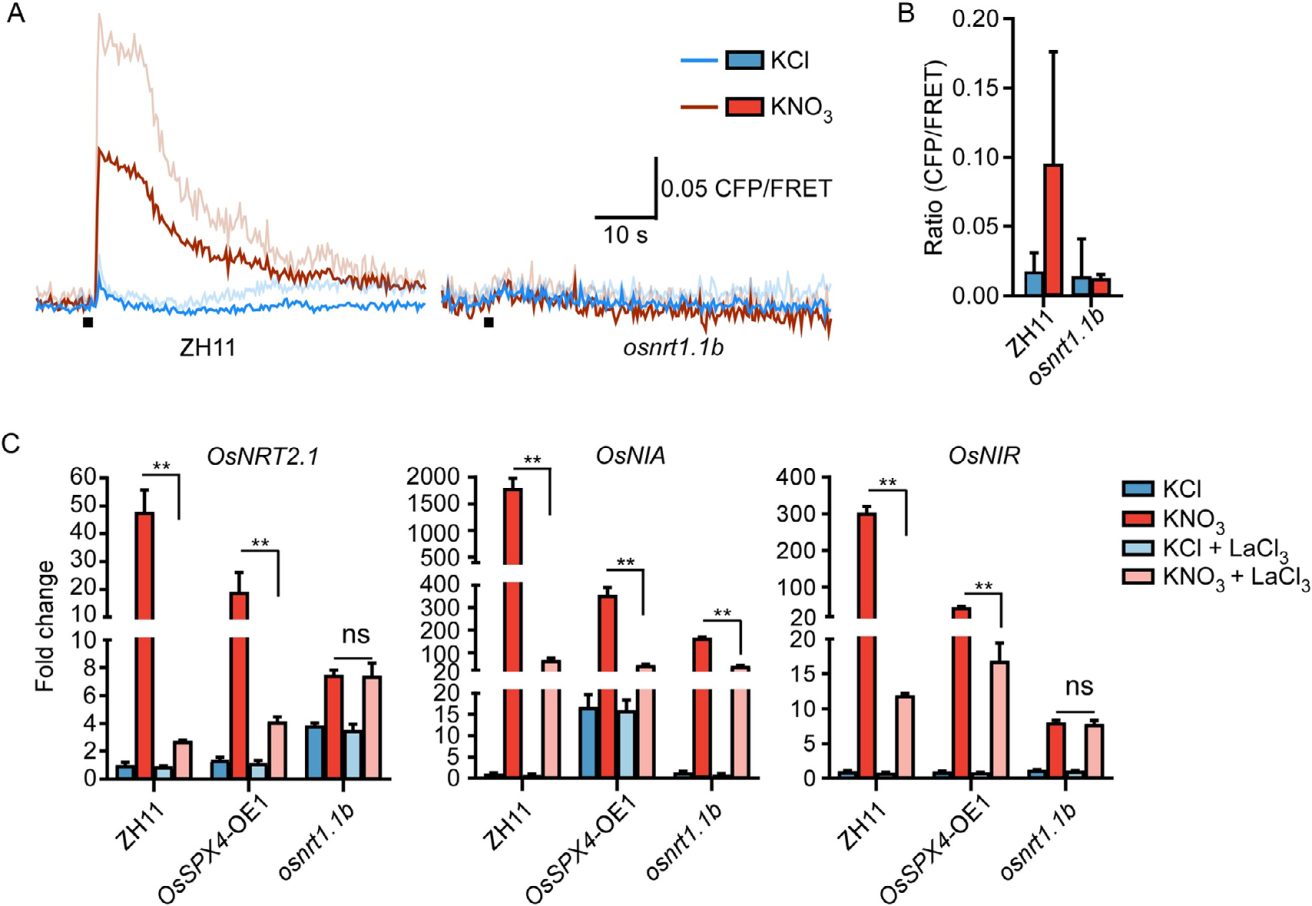
OsNRT1.1B mediates nitrate‐triggered Ca^2^⁺ signaling to complement the ubiquitination cascade in nitrate signaling A). Time‐course analysis of KNO_3_‐induced cytosolic Ca^2^⁺ ([Ca^2^^+^]_cyt_) dynamics. Root tips of ZH11 and *osnrt1.1b* plants expressing the Ca^2^⁺ sensor YC3.6 were treated with 10 mm KNO_3_ and 10 mm KCl served as a negative control. Images were acquired at 1‐s intervals. Black bars indicate the time‐points of when treatment was applied. Means ± SD. (*n* = 3 biological replicates). B). Quantification of nitrate‐triggered [Ca^2^^+^]_cyt_ elevation. Histogram indicates the average [Ca^2^^+^]_cyt_ levels in ZH11 and *osnrt1.1b* root tips treated by KNO_3_ or KCl. Means ± SD. (*n* = 3 biological replicates). C). Calcium channel blockade suppresses expression of primary nitrate‐responsive genes. RT‐qPCR analysis of nitrate‐inducible genes (*OsNRT2.1*, *OsNIA*, and *OsNIR*) in 14‐day‐old ZH11 and *OsSPX4‐*OE1 roots treated with 5 mm KNO_3_ for 30 min, with or without La^3^⁺ (Ca^2^⁺ channel inhibitor) pretreatment. Relative expression levels were normalized to the KCl‐treated ZH11 (control set as 1). Means ± SD. (*n* = 3 biological replicates; two‐tailed student's *t*‐test, ^**^ *P < 0.01*; ns, no significance).

In *Arabidopsis*, it was reported that the dual‐function “transceptor” AtNRT1.1 mediates nitrate‐induced Ca^2^⁺ influx,^[^ ^16^, ^20^, ^30^ ^]^ however, the role of calcium signaling in rice and its dependence on OsNRT1.1B remain unresolved. To investigate this, we used Zhonghua11 (ZH11) carrying the calcium indicator YC3.6 (ZH11^YC3.6^) to cross with the *osnrt1.1b* mutant, producing the *osnrt1.1b* ^YC3.6^. Application of 10 mm KNO_3_ to root tips elicited robust Ca^2^⁺ spikes in ZH11^YC3.6^ but not in *osnrt1.1b* ^YC3.6^ (**Figure** **1**A,B), demonstrating that OsNRT1.1B is essential for nitrate‐induced Ca^2+^ signaling in rice.

Given the critical role of Ca^2^⁺ in PNR activation, we further analyzed nitrate‐responsive gene expression, RT‐qPCR revealed strong suppression of *OsNIA*, *OsNIR*, and *OsNRT2.1* in *OsSPX4‐*OE1 and *osnrt1.1b* plants within 30 min of nitrate treatment (Figure 1C). Importantly, pretreatment with La^3^⁺, a plasma membrane Ca^2^⁺ channel blocker, significantly inhibited gene induction in ZH11 and *OsSPX4‐*OE1 plants but had minimal effect on *osnrt1.1b* mutant (Figure 1C). These findings indicate that both Ca^2+^ signaling and OsSPX4 degradation converge on OsNRT1.1B to orchestrate primary nitrate responses.

### 2.2 OsCNGC14/16 and OsNRT1.1B form a Nitrate‐Activated Calcium Channel

Cation channels mediating Ca^2+^ influx in plants belong to multiple protein families.^[^ ^31^ ^]^ Based on evidence that AtCNGC15 facilitates nitrate‐triggered Ca^2+^ signaling in *Arabidopsis* ^[^ ^16^ ^]^ and OsCNGCs enhance rice growth and yield,^[^ ^32^ ^]^ we systemically screened all 16 members of OsCNGC family to identify candidates mediating nitrate‐induced Ca^2+^ influx in rice. Using split‐ubiquitin yeast two‐hybrid (Y2H) assays, we identified interactions between OsNRT1.1B and OsCNGC1, 6, 10, 14, and 16 (Figure S2A, Supporting Information). RNA‐seq analysis of roots treated with 5 mm KNO_3_ showed nitrate‐inducible expression of *OsCNGC7*, *8*, *14*, and *16* (Figure S2B, Supporting Information). Furthermore, hydroponic assays under varying N regimes identified *OsCNGC3*, *5*, *8*, *13*, *14*, *15*, and *16* as being dynamically regulated in roots or shoots (Figure S2C, Supporting Information). Significantly, *OsCNGC14* and *16* consistently interacted with OsNRT1.1B, exhibited nitrate‐responsive expression in both short‐ and long‐term treatments, and showed co‐expression patterns with *OsNRT1.1B* (Figure S2D–F, Supporting Information). These results collectively suggest OsCNGC14 and OsCNGC16 as prime candidates for mediating nitrate‐triggered Ca^2^⁺ influx in rice.

**Figure 2:**
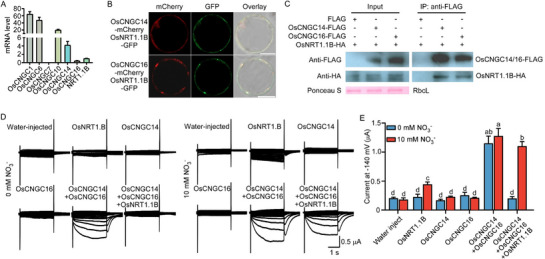
OsNRT1.1B‐OsCNGC14/16 complex functions as a nitrate‐responsive calcium channel A). Tissue‐specific expression of *OsCNGC* genes and *OsNRT1.1B* in rice root tips (≤5 mm). RT‐qPCR analysis of *OsCNGC1/6/7/10/14/16* and *OsNRT1.1B* expression in ZH11 root tips. Relative transcript levels were normalized to the expression level of *OsNRT1.1B* (set as 1). Means ± SD. (*n = 3* biological replicates). B). Subcellular co‐localization of OsCNGC14 and OsNRT1.1B, OsCNGC16 and OsNRT1.1B in rice protoplasts. Confocal images of 12‐day‐old rice protoplasts co‐expressing OsCNGC14‐mCherry and OsNRT1.1B‐GFP (top) or OsCNGC16‐mCherry and OsNRT1.1B‐GFP (bottom). Left, mCherry (561 nm excitation), middle, GFP (488 nm excitation), right, overlay (mCherry, GFP, and DIC) of the same sample. Scale bars = 10 µm. C). OsCNGC14 and OsCNGC16 physically interact with OsNRT1.1B in *planta*. Co‐immunoprecipitation (Co‐IP) assay in rice protoplasts co‐expressing OsCNGC14‐FLAG and OsNRT1.1B‐HA, OsCNGC16‐FLAG and OsNRT1.1B‐HA; FLAG and OsNRT1.1B‐HA was used as negative control. The ponceau S staining of Rubisco verifies equal protein loading in the input group. D). Nitrate‐regulated calcium channel activity of the OsNRT1.1B‐OsCNGC14/16 complex. Two‐electrode voltage‐clamp (TEVC) recordings from *Xenopus laevis* oocytes expressing *OsNRT1.1B*, *OsCNGC14*, *OsCNGC16*, *OsCNGC14* + *OsCNGC16*, and *OsCNGC14* + *OsCNGC16* + *OsNRT1.1B*. Bath solutions containing 20 mm CaCl_2_ + 10 mm KCl or 20 mm CaCl_2_ + 10 mm KNO_3_ (nitrate‐stimulated), by step‐wise model. Voltage steps: −140 to +20 mV (step 10 mV). Water‐injected was used as a negative control. E). Quantitative analysis of nitrate‐responsive calcium currents. Current amplitudes at −140 mV from multiple recordings in (D). Means ± SD. (*n* = 4 oocytes per group, one‐way ANOVA with Tukey's multiple comparisons test, *P < 0.05*).

To determine whether OsCNGC14, OsCNGC16, and OsNRT1.1B form a functional complex akin to *Arabidopsis* AtNRT1.1‐AtCNGC15 “transceptor‐channel”,^[^ ^16^ ^]^ we further analyzed their spatial and physical interactions. RT‐qPCR analysis confirmed overlapping expression of *OsCNGC14*, *OsCNGC16*, and *OsNRT1.1B* in root tips (**Figure** **2**A). They also co‐localized at the plasma membrane (Figure 2B), and Co‐IP and luciferase complementation imaging (LCI) assays further validated their interaction in *planta* (Figure 2C; Figure S3A, Supporting Information). Two‐Electrode Voltage Clamp (TEVC) recordings in *Xenopus* oocytes revealed that neither OsCNGC14 nor OsCNGC16 alone exhibited Ca^2^⁺ channel activity; however, when co‐expressed at a 1:1 ratio, they formed a functional heteromeric channel generating robust Ca^2^⁺ currents (Figure 2D,E). LCI and split‐ubiquitin Y2H assays confirmed heteromerization of OsCNGC14 and OsCNGC16 (Figure S3B,C, Supporting Information), which were co‐localized in oocyte membranes (Figure S3D, Supporting Information). Strikingly, co‐expression of *OsNRT1.1B* with *OsCNGC14* and *OsCNGC16* at a 1:1:1 ratio abolished these Ca^2^⁺ currents (Figure 2D,E), demonstrating that OsNRT1.1B inhibits the OsCNGC14/16 channel complex. Furthermore, ^15^N‐nitrate uptake assays confirmed that OsCNGC14 or OsCNGC16 neither facilitates nitrate transport nor influences the transport activity of OsNRT1.1B in oocytes (Figure S3E,F, Supporting Information), thereby underscoring their exclusive function in Ca^2^⁺ signaling.

Similar to the dual‐function nitrate sensor AtNRT1.1, OsNRT1.1B suppresses OsCNGC14/16 activity, forming a resting transceptor‐channel complex (Figure 2D,E). To examine whether nitrate perception influences this interaction, we treated oocytes co‐expressing *OsNRT1.1B* and *OsCNGC14/16* with 10 mm nitrate. Nitrate treatment restored Ca^2^⁺ currents of OsNRT1.1B‐OsCNGC14/16 to levels approaching those observed with OsCNGC14/16 (Figure 2D,E). Consistent with this result, Co‐IP assays in rice protoplasts expressing *OsCNGC14‐FLAG* and *OsNRT1.1B‐HA, OsCNGC16‐FLAG* and *OsNRT1.1B‐HA* showed a strong basal interaction that was weakened upon nitrate treatment (Figure S3G, Supporting Information); LCl assays in tobacco leaves confirmed this result (Figure S3H,I, Supporting Information). Together, these findings define a nitrate‐triggered calcium channel formed by the heteromeric OsCNGC14/16 complex and OsNRT1.1B. Nitrate perception relieves OsNRT1.1B‐mediated inhibition, thereby activating the channel and initiating Ca^2^⁺ influx.

### 2.3 OsCNGC14 and OsCNGC16 are Essential for Nitrate‐Induced Calcium Signaling and Nitrogen Utilization in Rice

**Figure 3:**
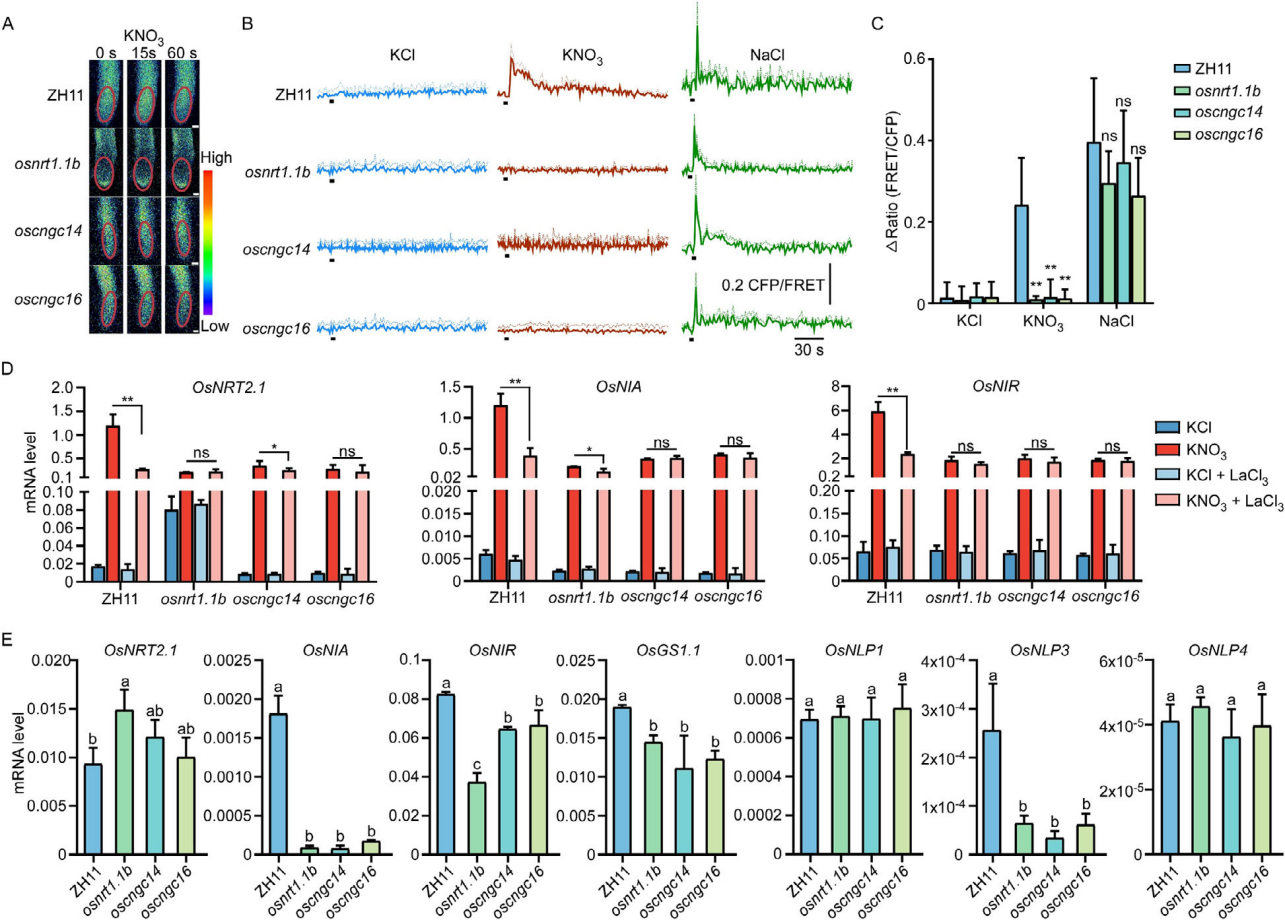
The *oscngc14* and *oscngc16* display abolished Ca^2+^‐dependent PNR and reduced long‐term expression of nitrogen assimilation genes. A). Images of nitrate‐triggered cytosolic Ca^2^⁺ ([Ca^2^^+^]_cyt_) in root tips. Time‐lapse pseudo‐color ratio imaging of [Ca^2^^+^]_cyt_ in ZH11, *oscngc14*, and *oscngc16* root tips were treated with 10 mm KNO_3_. Time‐point of 0 s indicates treatment initiation. Red circles denote regions of root tips used for ratiometric measurements. The [Ca^2^^+^]_cyt_ responses were visualized using a color gradient from low (blue) to high (red). Scale bar = 50 µm. B). Time‐course analysis of nitrate‐induced [Ca^2^^+^]_cyt_ fluxes. ZH11, *osnrt1.1b*, *oscngc14*, and *oscngc16* root tips expressing the Ca^2+^ sensor YC3.6 were treated with 10 mm KCl (left, negative control, *n = 3* biological replicates), 10 mm KNO_3_ (middle, *n = 4* biological replicates), and 100 mm NaCl (right, osmotic control, *n = 3* biological replicates). Images were acquired at 1 s intervals. Black bar indicates the time‐points of treatment applied. Means ± SD. C). Quantitative analysis of [Ca^2^^+^]_cyt_ responses. Histogram indicates the average [Ca^2^^+^]_cyt_ of ZH11, *osnrt1.1b*, *oscngc14*, and *oscngc16* root tips treated by KCl, KNO_3_, and NaCl in (B). Means ± SD. (*n ≥ 3* biological replicates, two‐tailed Student's *t*‐test ** *P < 0.01*; ns, no significance). D). The mutants of *OsCNGC14* and *OsCNGC16* blockade suppresses expression of primary nitrate‐responsive genes. RT‐qPCR analysis of primary nitrate‐inducible genes (*OsNRT2.1*, *OsNIA*, and *OsNIR*) in 14‐day‐old ZH11, *osnrt1.1b*, *oscngc14*, and *oscngc16* roots treated with 10 mm KNO_3_ or KCl for 30 min, with or without LaCl_3_ pretreatment. Means ± SD. (*n = 3* biological replicates; two‐tailed student's *t*‐test, ^*^ *P < 0.05*, ** *P < 0.01*; ns, no significance). E). RT‐qPCR analysis of N‐related genes (*OsNRT2.1*, *OsNIA*, *OsNIR, OsGS1.1, OsNLP1, OsNLP3*, and *OsNLP4*) in 60‐day‐old of ZH11, *osnrt1.1b*, *oscngc14*, and *oscngc16* roots which were grown in greenhouse. Means ± SD. (*n = 3* biological replicates sample; one‐way ANOVA with Tukey's multiple comparisons test, *P < 0.05*).

To investigate whether OsCNGC14 and OsCNGC16 are involved in nitrate‐induced calcium signaling, we generated loss‐of‐function mutants, *oscngc14* and *oscngc16*, using the CRISPR/Cas9 system (Figure S4, Supporting Information). These mutants were subsequently crossed with the ZH11^YC3.6^ to produce the *oscngc14* ^YC3.6^ and *oscngc16* ^YC3.6^ lines. Nitrate‐triggered Ca^2^⁺ influx was monitored in root tips by treating with 10 mm KNO_3_. A robust calcium spike was observed in ZH11^YC3.6^ roots, but not in *oscngc14* ^YC3.6^, *oscngc16* ^YC3.6^, and *osnrt1.1b* ^YC3.6^ lines (**Figure** **3**A–C). Notably, NaCl‐induced Ca^2^⁺ influx remained unaffected in all lines (Figure 3B,C), demonstrating that OsCNGC14 and OsCNGC16 are essential for generating nitrate‐triggered elevations in intracellular calcium concentration ([Ca^2^^+^]_i_) in root tip cells.

Next, we assessed the role of OsCNGC14 and OsCNGC16 in Ca^2^^+^‐dependent nitrate‐responsive gene expression using RT‐qPCR analysis. In roots, the expression of key target genes, *OsNRT2.1*, *OsNIA*, and *OsNIR*, was reduced in *osnrt1.1b*, *oscngc14*, and *oscngc16* mutants (Figure 3D). These reductions mirrored the effect observed in ZH11 roots pretreated with the Ca^2^⁺ channel blocker LaCl_3_. Under infertile‐soil culture in a greenhouse, N assimilation genes (*OsNIA*, *OsNIR*, *OsGS1.1*) and the transcription factor *OsNLP3* were significantly downregulated in *osnrt1.1b*, *oscngc14*, and *oscngc16* mutants, while other nitrate‐responsive transcription factors remained unchanged (Figure 3E). Combined with previous reports of growth inhibition in *osnlp3* mutant,^[^ ^27^ ^]^ these findings suggest that calcium‐dependent PNR may regulate long‐term expression of N‐related genes in rice via *OsNLP3* expression.

Field trials conducted under low‐ and normal‐N conditions demonstrated severe growth defects in *oscngc14* and *oscngc16* mutants, including reduced plant height, panicle length, tiller number, and ultimately lower grain yield compared to ZH11 under both N regimes, which was similar to the *osnrt1.1b* mutant (Figure S5A–C, Supporting Information). Hydroponic assays further confirmed impaired shoot development, root architecture establishment, and reduced biomass accumulation in *osnrt1.1b*, *oscngc14*, and *oscngc16* mutants under insufficient and sufficient N supplies (Figure S5D,E, Supporting Information). Moreover, ^15^N‐nitrate or ^15^N‐ammonium feeding experiments showed reduced nitrate uptake and root‐to‐shoot transport in these mutants, while ammonium utilization was less affected. This reduction ultimately resulted in a decrease of total N content in both roots and shoots (Figure S5F,G, Supporting Information). Coupled with the altered expression of nitrogen‐related genes (Figure 3E), this disturbance of N homeostasis led to reduced grain yield and NUE in *osnrt1.1b*, *oscngc14*, and *oscngc16* mutants. Notably, *OsSPX4*‐OE1 and *osnbip1*‐*1* plants exhibited PNR repression similar to that of *osnrt1.1b*, *oscngc14*, and *oscngc16* mutants (Figures 1, 3; Figure S1, Supporting Information). However, unlike these mutants, *OsSPX4‐*OE and *osnbip1* plants showed only slight reductions in shoot growth, root development, biomass, N uptake, and N content in hydroponic or ^15^N feeding experiments (Figure S6A–D, Supporting Information). Furthermore, *OsSPX4*‐OE1 plants showed no significant growth inhibition in the field, particularly in tiller number (Figure S6E, Supporting Information). These results suggest that nitrate‐induced Ca^2^⁺ signaling regulates both PNR and long‐term nitrate responses through a pathway distinct from the OsSPX4 degradation cascade.

### 2.4 Phosphorylation of OsNLP3 at Ser193 Links Nitrate‐Induced Calcium Signaling and Transcriptional Activation

OsNLP3, a key transcription factor, is released from OsSPX4 repression upon nitrate exposure.^[^ ^13^ ^]^ However, it remains unclear whether OsNLP3 undergoes nitrate‐induced activation via phosphorylation—similar to AtNLP7/6 in *Arabidopsis*.^[^ ^15^ ^]^ To address this, we utilized *OsNLP3‐FLAG* transgenic plants to test OsNLP3 protein modification in phos‐tag assays. Interestingly, it revealed that nitrate triggers the phosphorylation of OsNLP3, as the effect was abolished by λ phosphatase treatment (Figure S7A, Supporting Information). Furthermore, pretreatment with LaCl_3_ dramatically reduced this phosphorylation (Figure S7B, Supporting Information). Sequence alignment identified a conserved phosphorylation residue at serine 193 (Ser193)^[^ ^15^ ^]^ within the GAF domain across NLPs in both *Arabidopsis* and rice (Figure S7C, Supporting Information). This residue was also predicted by prediction tools (NetPhos‐3.1). Mutational analysis further confirmed the functionality of this residue: a phosphoablative variant (OsNLP3^S193A^) exhibited attenuated phosphorylation, whereas a phosphomimetic variant (OsNLP3^S193D^) displayed constitutive phosphorylation (Figure S7D, Supporting Information). Together, these findings establish Ser193 as the primary residue of nitrate‐induced phosphorylation.

Next, we investigated how phosphorylation influences OsNLP3 activity by examining its subcellular localization in rice protoplasts. Following nitrate stimulation, both eGFP‐OsNLP3 and eGFP‐OsNLP3^S193D^ rapidly translocated to the nucleus within 45 min. In contrast, the eGFP‐OsNLP3^S193A^ showed delayed nuclear accumulation, requiring 90 min (Figure S8A, Supporting Information). This observation was confirmed by protein degradation assays and nuclear‐cytoplasmic fractionation (Figure S8B,C, Supporting Information), suggesting that phosphorylation at Ser193 accelerates the nuclear translocation of OsNLP3. Luciferase assays demonstrated that OsNLP3^S193D^ enhanced transcriptional activation of key target genes, *OsNRT2.1*, *OsNIA*, and *OsGS1.1*, while OsNLP3^S193A^ increased *OsNIR* expression (Figure S9A, Supporting Information). Importantly, both the phosphoablative and phosphomimetic variants of OsNLP3 acted as positive regulators of target genes. Moreover, when complemented in *osnlp3* protoplasts, all three forms (OsNLP3‐FLAG, OsNLP3^S193A^‐FLAG, or OsNLP3^S193D^‐FLAG) showed comparable protein abundance (Figure S10, Supporting Information) and similarly upregulated target genes (Figure S9B, Supporting Information). These findings indicate that although phosphorylation of Ser193 enhances OsNLP3 nuclear translocation or transcriptional activity, it is not essential for these processes. This provides regulatory flexibility critically for rice adaptation to fluctuating nitrate availability, unlike the mandatory requirement seen for AtNLP7 activation.

**Figure 4:**
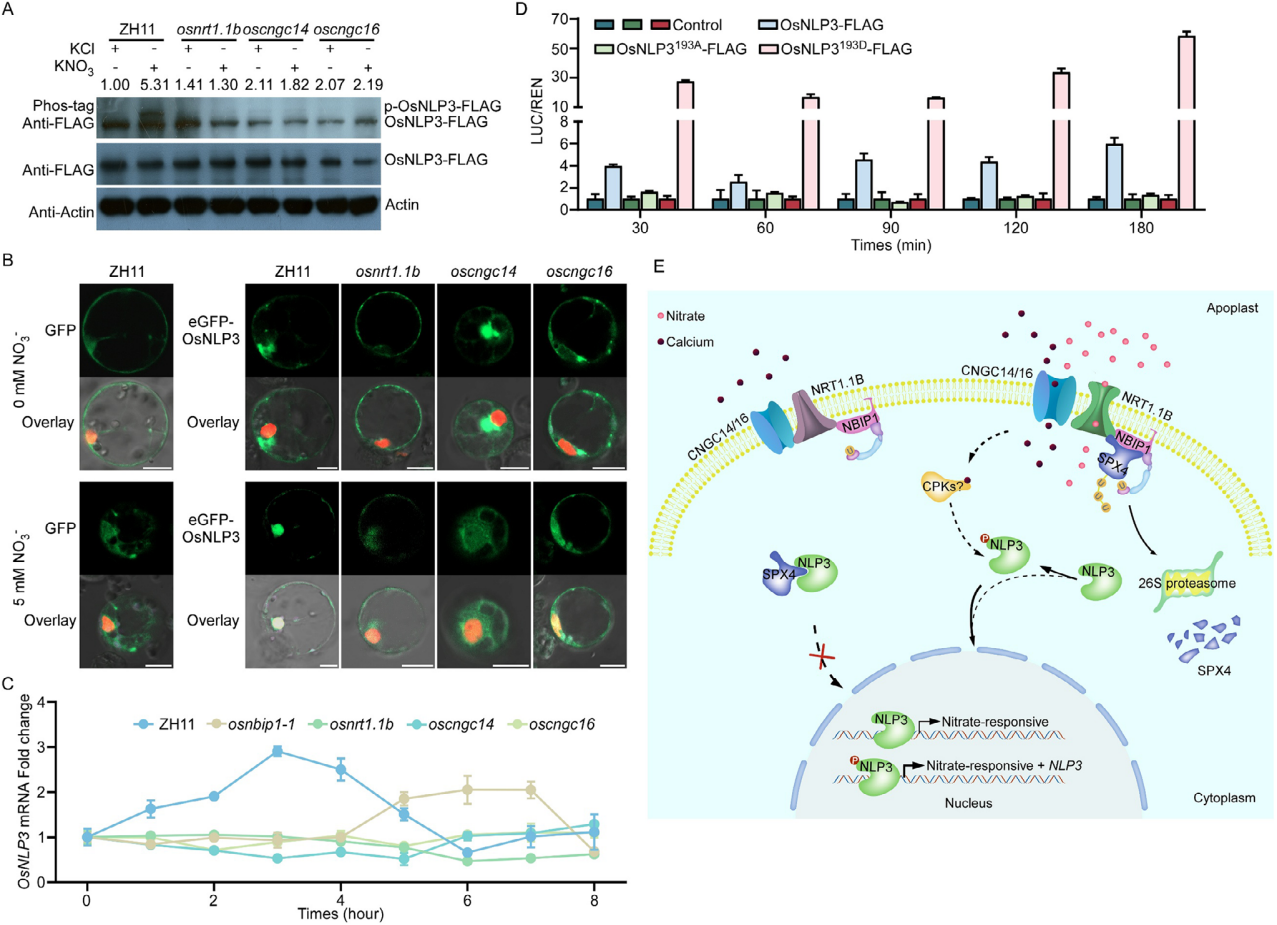
OsNRT1.1B‐OsCNGC14/16‐mediated phosphorylation triggers accelerated nuclear translocation and enhances transcriptional activation of *OsNLP3*. A) Nitrate‐induced OsNLP3 phosphorylation depends on OsNRT1.1B, OsCNGC14, and OsCNGC16. Phos‐tag and SDS‐PAGE analysis of OsNLP3‐FLAG phosphorylation in protoplasts from ZH11, *osnrt1.1b*, *oscngc14*, and *oscngc16* plants treated with 10 mm KNO_3_ or KCl for 30 min. Phosphorylation intensity (normalized to KCl‐treated ZH11, set as 1) was quantified from 8% phos‐tag gels (top). Total protein levels were verified by 10% SDS‐PAGE (middle). Actin serves as loading control (bottom). B) OsNRT1.1B‐OsCNGC14/16 accelerates nuclear translocation of OsNLP3 under nitrate treatment. Confocal images of protoplasts co‐expressing e*GFP‐OsNLP3* and *HY5‐mCherry* from 12‐day‐old ZH11, *osnrt1.1b*, *oscngc14*, and *oscngc16* plants. The protoplasts were treated with 5 mm KNO_3_ or KCl for 45 min. GFP fluorescence (488 nm excitation) (top). Overlay (GFP, mCherry and DIC) of the same sample (bottom). Scale bars = 5 µm. C) Calcium signaling sustains *OsNLP3* transcriptional activation. Time‐course RT‐qPCR of *OsNLP3* expression in ZH11, *osnbip1‐1*, *osnrt1.1b*, *oscngc14*, and *oscngc16* roots treated with 5 mm KNO_3_ or KCl for 0–8 h. Data are normalized to KCl‐treated controls (set as 1). Means ± SD. (*n = 3* biological replicates). D). Phosphorylation of OsNLP3 at Ser193 confers OsNLP3 transcriptional activation. Transactivation assays of OsNLP3‐FLAG, OsNLP3^S193A^‐FLAG or OsNLP3^S193D^‐FLAG with the promoters of *OsNLP3* were conducted. The dual‐luciferase reporter plasmids driven by *OsNLP3* promoter were transiently expressed in os*nlp3* protoplasts together with empty vector (control) or OsNLP3 effectors (OsNLP3‐FLAG, OsNLP3^S193A^‐FLAG, or OsNLP3^S193D^‐FLAG), respectively. Luciferase activity was measured at 30–180 min after treatment with 5 mM KNO_3_. Data are normalized to OsNLP3‐FLAG control (set as 1). Means ± SD. (*n = 3* biological replicates). E). Nitrate signaling network integrates with calcium signaling and canonical ubiquitination cascade in rice. (1) OsNRT1.1B‐OsCNGC14/16‐Ca^2^⁺ pathway: nitrate‐triggered calcium influx phosphorylates OsNLP3 (Ser193), enabling its rapid nuclear translocation and higher transcriptional autoactivation. (2) OsNRT1.1B‐OsNBIP1‐OsSPX4 pathway: ubiquitination‐mediated OsSPX4 degradation releases OsNLP3 for sustained N assimilation.

Consistent with disrupted Ca^2^⁺ signaling in *osnrt1.1b*, *oscngc14*, and *oscngc16* mutants (Figure 3B), we also observed abolished OsNLP3 phosphorylation and delayed nuclear translocation in these mutants (**Figure** **4**A,B), directly linking Ca^2^⁺ signaling to OsNLP3 activation. While *OsNLP3* transcript levels increased 2 h after N starvation following nitrate resupply,^[^ ^27^ ^]^ this induction was absent in *osnrt1.1b*, *oscngc14*, and *oscngc16* mutants, with only a delayed response observed in the *osnbip1* mutant (Figure 4C). Transactivation assays revealed OsNLP3^S193D^ strongly activated its own promoter, whereas the OsNLP3^S193A^ variant showed minimal activity (Figure 4D). This result indicates that Ca^2^⁺ signaling is essential for activating the transcription of *OsNLP3* in response to nitrate. Accordingly, RT‐qPCR verified that nitrate‐responsive genes were not activated in the roots of *osnrt1.1b*, *oscngc14*, and *oscngc16* mutants within 4–6 h of treatment (Figure S11, Supporting Information). In summary, our results show that nitrate‐triggered calcium influx starts a signaling cascade that leads to the phosphorylation of OsNLP3 at Ser193, allowing its self‐activation and promoting the expression of nitrate‐responsive genes. (Figure 4E).

## 3 Discussion

Plants have evolved distinct nitrate signaling pathways to adapt to fluctuating N availability, as illustrated by the OsNRT1.1B‐OsNBIP1‐OsSPX4‐OsNLP3 pathway in rice and the AtNRT1.1‐Ca^2+^‐AtCPKs‐AtNLP7 pathway in *Arabidopsis*.^[^ ^13^, ^15^, ^16^ ^]^ Comparative analyses of these systems provide critical insights into the evolutionary divergence of nitrate‐sensing mechanisms. In this study, we identified the OsNRT1.1B‐OsCNGC14/16 complex as a dual‐function transceptor‐channel that senses elevated nitrate levels and triggers calcium influx to initiate downstream signaling (Figure 4E).

To dissect the roles of these pathways, we compared the growth phenotypes and N homeostasis of related mutants and overexpression plants in two signaling pathways under low and normal N conditions. Although the two pathways regulate different developmental processes and aspects of N homeostasis, the adverse effects of disrupted nitrate signaling on growth and N regulation become less severe under higher N conditions (Figures S5 and S6, Supporting Information). These findings suggest that elevated nutrient levels attenuate the influence of nitrate signaling on growth and development, especially in root architecture establishment. This mechanism explains how the yield penalty is alleviated in *oscngc14* and *oscngc16* mutants under normal N supply (Figure S5A,B, Supporting Information). Our results imply that enhancing nitrate signaling could significantly improve NUE in N‐limited soils.

Notably, rice appears to employ more complex layers of regulation than *Arabidopsis*. While calcium signaling is essential for sustained nitrate responses, OsNLP3 retains partial Ca^2^⁺‐independent activity, suggesting that rice integrates both canonical and lineage‐specific mechanisms in nitrate signaling. Rather than substituting the canonical ubiquitination pathways in rice, Ca^2+^ signaling collaborates with the OsSPX4‐mediated repression system, thereby facilitating the precise regulation of N metabolism under fluctuating environmental conditions. This dual‐layer regulation, combining rapid Ca^2^⁺‐dependent phosphorylation with the adjustable interactions between OsSPX4 and OsNLP3, may underpin rice's exceptional adaptability to fluctuating nutrient availability.

Beyond their role in N signaling, OsCNGCs affect plant growth and responses to various abiotic stresses, including ABA signaling, reactive oxygen species (ROS), and temperature fluctuations.^[^ ^33^, ^34^, ^35^, ^36^ ^]^ Intriguingly, OsCNGC14 and OsCNGC16 participate in both ABA and temperature signaling and nitrate‐triggered Ca^2^^+^ influx.^[^ ^35^, ^36^ ^]^ Although the hypersensitivity of the *oscngc14/16* double mutant to cold suggests functional redundancy between the two members in temperature signaling,^[^ ^35^ ^]^ the molecular basis for their interaction remains unclear. Our evidence indicates that OsCNGC14 and OsCNGC16 form a heteromeric complex specifically responsible for nitrate‐triggered calcium influx (Figure 2D,E, and 3A‐C). Mechanistically, these channels appear to operate via different activation modes: 1) In temperature signaling, they may function similarly to AtCNGC5 and AtCNGC6 in the cold response,^[^ ^37^ ^]^ potentially exhibiting functional redundancy; 2) In nitrate signaling, they resemble AtCNGC15,^[^ ^16^ ^]^ working cooperatively with AtNRT1.1 to form a transceptor‐channel complex for nitrate‐triggered calcium signal encoding. Therefore, our discovery of the OsNRT1.1B‐OsCNGC14/16 interaction establishes a direct mechanistic link between nutrient availability and stress resistance.

Among the 16 OsCNGCs in rice, only a subset, including OsCNGC14 and OsCNGC16, has defined roles in signaling or ion transport.^[^ ^33^, ^34^, ^35^, ^36^, ^38^ ^]^ Our investigation reveals that multiple OsCNGCs associate with OsNRT1.1B, although their functional relevance remains to be characterized. Given the established role of OsNRT1.1B in nitrate/phosphate signaling and ammonium utilization, these associated OsCNGCs may mediate additional processes such as auxin transport or root development, as seen in *Arabidopsis*.^[^ ^39^ ^]^ Future studies should explore whether OsCNGCs act as versatile signaling hubs that coordinate nutrient sensing with developmental processes.

In conclusion, our data delineate two interconnected pathways governing nitrate responses in rice: 1) The OsNRT1.1B‐OsNBIP1‐OsSPX4‐OsNLP3 axis fine‐tunes N metabolism in response to short‐term nitrate fluctuations (minutes to 2–3 h), ensuring a baseline toward N supply; 2) The OsNRT1.1B‐OsCNGC14/16‐Ca^2^⁺‐OsNLP3 pathway enables rapid detection (seconds to minutes) of external nitrate surges and sustains long‐term N homeostasis (3–4 h to weeks) through phosphorylation‐mediated mechanisms. These pathways allow rice to dynamically balance immediate nutrient acquisition with long‐term metabolic adjustments. Our work not only advances mechanistic understanding of nitrate signaling but also identifies conserved nodes at the intersection of nutrient sensing and stress response as promising targets for improving crop resilience.

## 4 Experimental Section

### Plant Materials and Growth Conditions

The wild‐type rice Zhonghua 11 (*O. sativa* L. *japonica*, ZH11) was used in this study. The mutants of *osnrt1.1b*, *osnbip1‐1*, *osnbip1‐2*, and *osnlp3*, as well as the overexpression lines of *OsSPX4‐*OE1, *OsSPX4‐*OE2, and *OsNLP3‐FLAG‐*OE were previously generated in the laboratory. The *oscngc14* and *oscngc16* mutants were generated in the ZH11 background using *CRISPR/Cas9*‐mediated gene editing. For cytosolic imaging assays, *osnrt1.1b, oscngc14*, and *oscngc16* mutants were crossed with YC3.6‐expressing plants (ZH11^YC3.6^). For hydroponic culture (1–3 weeks; used for nitrate induction experiments, gene expression analysis, and rice protoplast preparation), rice seedlings were grown in a growth chamber with a 16‐h light/8‐h dark photoperiod, light intensity of ≈200 µmol m^−2^ s^−1^, temperature of 28/25 °C (day/night), and humidity of ≈70%. The basic nutrient solution contained the following macronutrients (in mm): (NH_4_)_2_SO_4_ (0.5), MgSO_4_·7H_2_O (0.54), CaCl_2_·2H_2_O (0.36), K_2_SO_4_ (0.1), KH_2_PO_4_ (0.18), and Na_2_SiO_3_·9H_2_O (1.6). The micronutrient composition (in µm) was: MnCl_2_·4H_2_O (9.14), H_3_BO_3_ (46.2), (NH_4_)_6_Mo_7_O_24_·4H_2_O (0.08), ZnSO_4_·7H_2_O (0.76), CuSO_4_·5H_2_O (0.32), and Fe(II)‐EDTA (40), adjusted to pH 5.7. The nutrient solution was replaced daily. Different concentrations of KNO_3_ were added to the basic solution depending on experimental treatments. For long‐term greenhouse cultivation (2–3 months; used for detecting long‐term root N‐responsive gene expression), rice plants were grown in a greenhouse under low N condition: 5 g of N (mixture of 60% nitrate, 40% ammonium) per 1 m^2^ with a 12‐h light/12‐h dark photoperiod, natural light intensity, temperature of 30/24 °C (day/night), and humidity of ≈40–60%.

### Vector Construction and Generation of Transgenic Rice

To generate *oscngc14* and *oscngc16* mutants, the 20‐nt small guiding RNA was cloned into *CRISPR/Cas‐BGK03* (Biogle).^[^ ^40^ ^]^ Then the vectors were introduced into the *Agrobacterium* strain EHA105. The wild‐type ZH11 was used as the recipient for *Agrobacterium*‐mediated transformation, as previously described, to generate the transgenic rice.^[^ ^41^ ^]^ All constructs were verified by sequencing. T3 homozygous plants were used for subsequent experiments. For split‐ubiquitin analysis, the full‐length coding sequence (CDS) of *OsNRT1.1B* and *OsCNGCs* were cloned to generate either the *pPR3‐N* (*OsNRT1.1B*, *OsCNGC16*) or *pMET* (*OsCNGCs*). For studying protein interactions in *Nicotiana benthamiana* using *OsCNGC14‐nLUC*, O*sCNGC16*‐*nLUC*, *cLUC*‐*OsNRT1.1B*, and *cLUC*‐*OsCNGC16* constructs, CDS of *OsNRT1.1B*, *OsCNGC14*, and *OsCNGC16* were amplified and cloned into *pCAMBIA1300*‐*nLUC* and *pCAMBIA1300‐(ATG)‐cLUC* vectors. To observe co‐immunoprecipitation (Co‐IP) in protoplasts and subcellular localization in protoplasts and *Xenopus* oocytes using *OsNRT1.1B‐GFP/HA*, *OsCNGC14‐mCherry/GFP/FLAG*, *OsCNGC16‐mCherry/FLAG, AtHY5‐mCherry* and *eGFP‐OsNLP3*, CDS of *OsNRT1.1B*, *OsCNGC14, OsCNGC16* from ZH11 and *AtHY5* from *Arabidopsis thaliana* ecotype Col‐0 were amplified and cloned into *pGEMHE‐eGFP, pGEMHE‐mCherry*, *pSAT1‐35S‐eGFP/mCherry*, *pCAMBIA2300‐35S‐HA*, *pCAMBIA2300*‐*35S‐FLAG*, and *pCAMBIA2300*‐*35S‐eGFP‐N* vectors. Site‐directed mutagenesis was used to generate *pCAMBIA2300*‐*35S‐eGFP‐OsNLP3^S193A^* and *pCAMBIA2300*‐*35S‐eGFP‐OsNLP3^S193D^* constructs. For electrophysiological assay in *Xenopus* oocytes using *OsNRT1.1B*, *OsCNGC14*, and *OsCNGC16*, CDS of *OsNRT1.1B*, *OsCNGC14*, and *OsCNGC16* from ZH11 were amplified and cloned into *pGEMHE* vectors. To examine OsNLP3 phosphorylation and the effects of phosphorylated/non‐phosphorylated OsNLP3 on N‐responsive gene activation, CDS sequences of *OsNLP3* and promoter sequences (2 kb upstream of ATG) of *pOsNRT2.1*, *pOsNIA1*, *pOsNIR1*, *pOsGS1.1*, and *pOsNLP3* were cloned into *pCAMBIA2300*‐*35S‐FLAG* and *pGreenII0800*‐*ubiq‐LUC* vectors. Site‐directed mutagenesis was used to generate *pCAMBIA2300*‐*35S‐OsNLP3^S193A^‐FLAG* and *pCAMBIA2300‐35S‐OsNLP3^S193D^‐FLAG* constructs.

### Nitrate Induction Assay of Nitrate‐Responsive Genes

Rice seedlings were grown in a basic nutrient solution containing 0.5 mm (NH_4_)_2_SO_4_ for 2 weeks. Prior to the nitrate Induction assay, seedlings were pretreated with 0.5 mm (NH_4_)_2_SO_4_ in basic solution for 48 h under continuous light (including the 2‐week growth period), and were pretreated with or without 2 mm LaCl_3_ for 10 min, then transferred to a fresh solution supplemented with 5 mm KNO_3_ or KCl. Roots were collected at specific time‐points for gene expression analysis (*n* = 3 replicates, 6–8 seedlings per replicate).

### Yeast Two‐Hybrid Assays

*pPR3‐N* (*OsNRT1.1B*, *OsCNGC16*) and *pMET* (*OsCNGCs*) vectors were introduced into yeast strain NMY51 using the lithium acetate method. The NMY51 transformants were selected on SD medium lacking Leu and Trp for 3 days at 28 °C. Then the haploid cells of NMY51 were selected on SD medium containing 5 mm 3‐AT, lacking Leu, Trp, His, and Ade for 5 days at 28 °C. The combinations in this experiment were N:APP/pMET:Fe65 as positive control; pPR3‐N:OsNRT1.1B/pMET‐EV and pPR3‐N:OsCNGC16/pMET‐EV as negative controls.

### RNA Isolation and qPCR Analysis

Total RNA was extracted using TRIzol reagent (Invitrogen) and reverse‐transcribed with ReverTra Ace qPCR RT Master Mix (Toyobo). qPCR was performed using SYBR Green Master Mix (Toyobo) on a Chromo4 system (Bio‐Rad). Data were analyzed with Excel (Microsoft). Rice *Actin1* served as the internal reference. Primers are listed in Table S1 (Supporting Information).

### RNA‐Seq Analysis

Approximately 100 ZH11 seedlings per treatment were hydroponically grown as described above. For nitrate induction, seedlings pretreated with 1 mm ammonium were exposed to 5 mm KNO_3_ or KCl. Samples were collected at 0, 1.75, 2.75, and 24 h (*n* = 3 replicates, 15 seedlings per replicate). For long‐term nitrate treatments, seedlings were cultured in modified Kimura B solution (0, 0.2, 2, and 10 mm KNO_3_) with adjusted potassium concentrations. Roots and shoots of 14‐day‐old seedlings were collected for RNA‐seq (*n* = 3 replicates, 15 seedlings per replicate). BGI‐Wuhan performed library construction and sequencing.

### Subcellular Localization Assay

All subcellular localization experiments used nitrate‐free rice protoplasts. Protoplasts were isolated from 12‐day‐old seedlings and transformed as previously described.^[^ ^42^ ^]^ To investigate the co‐localization of OsNRT1.1B with OCNGC14 or OsCNGC16. Nitrate‐free rice protoplasts were co‐transfected with *pSAT1*‐*35S‐OsNRT1.1B‐mCherry* and either *pSAT1*‐*35S‐OsCNGC14‐GFP* or *35S‐OsCNGC16‐GFP*. To study the effect of *osnrt1.1b*, *oscngc14*, and *oscngc16* on OsNLP3 localization under nitrate induction, nitrate‐free protoplasts were co‐transfected with *pCAMBIA2300‐35S*‐*eGFP‐OsNLP3* and *pSAT1‐35S*‐*AtHY5*‐*mCherry* (1:0.8 ratio). To test the effect of Ser193 on OsNLP3 localization under nitrate induction, nitrate‐free protoplasts were co‐transfected with *pCAMBIA2300‐35S*‐*eGFP‐OsNLP3*/*35S*‐*eGFP*‐*OsNLP3^S193A^*/*35S*‐*eGFP*‐*OsNLP3^S193D^* and *pSAT1‐35S*‐*AtHY5*‐*mCherry* (1:0.8 ratio). Incubate the rice protoplasts transfected with the plasmid in W5 solution for 12 h. Protoplasts were centrifuged at 200 × *g* for 3 min, treated with W5 solution containing 5 mm KNO_3_ for 0, 45, and 90 min, and observed under a confocal microscope (LSM 980, Carl Zeiss).

### Luciferase Complementation Imaging (LCI) Assay

LCI assays were conducted to study interactions between OsCNGC14‐nLUC/cLUC‐OsNRT1.1B, OsCNGC16‐nLUC/cLUC‐OsNRT1.1B, and OsCNGC14‐nLUC/cLUC‐OsCNGC16. *Agrobacterium* strain *GV3101* carrying constructs was cultured in LB medium at 28 °C for 16 h. Cells were pelleted, resuspended in infiltration buffer (10 mm MES (pH 5.6), 10 mm MgCl_2_, 0.2 mM acetosyringone; OD_600_ = 1.0), and incubated at room temperature for 3 h. Equal volumes of bacterial suspensions were co‐infiltrated into *N. benthamiana* leaves. Plants were kept at 23 °C for 48 h (16‐h light/8‐h dark). Luciferin (1 mm) and 0.01% Triton X‐100 (v/v) were sprayed onto leaves, and luminescence was captured using a low‐light CCD camera (NightOWL II LB983).

For LCI assays, the effect of nitrate on the interactions between OsNRT1.1B and OsCNGC14, OsNRT1.1B and OsCNGC16 was examined. The infiltrated leaves were first treated with 10 mm KNO_3_ or KCl for 30 min, and then treated as described above.

### Co‐Immunoprecipitation (Co‐IP) Assay

Nitrate‐free protoplasts co‐expressed with OsCNGC14‐FLAG + OsNRT1.1B‐HA and OsCNGC16‐FLAG + OsNRT1.1B‐HA were incubated in W5 solution for 12 h, centrifuged at 200 × *g*, and treated with 10 mm KNO_3_ or KCl for 30 min. Total proteins were extracted from lysed protoplasts using 200 µL extraction buffer (50 mm HEPES (pH 7.5), 150 mm NaCl, 1% (v/v) Triton X‐100, 10% (v/v) glycerol, 1× EDTA‐free protease inhibitor cocktail). A 20 µL aliquot was mixed with 5 µL 6× SDS loading buffer as input. The remaining lysate was diluted to 1 mL with extraction buffer (without Triton X‐100), incubated with 20 µL anti‐FLAG magnetic beads (Lablead PFM050) at 4 °C for 2 h. Beads were washed three times with buffer (50 mm HEPES (pH 7.5), 150 mm NaCl), eluted with 2× SDS loading buffer at 55 °C for 10 min, and analyzed by SDS‐PAGE and immunoblotting using HRP‐conjugated anti‐FLAG or anti‐HA antibodies.

### Two‐Electrode Voltage Clamp (TEVC) and Xenopus Oocyte ^15^N Uptake Assay

Full‐length CDS of *OsCNGC14*, *OsCNGC16*, and *OsNRT1.1B* were cloned into *pGEMHE*. Capped RNAs (cRNAs) were synthesized using the mMACHINE T7 Kit (Thermo) and stored at −80 °C. Stage V‐VI *Xenopus* oocytes were defolliculated with collagenase A (1.5 mg mL^−1^ in Ca^2^⁺‐free ND96) at 26 °C, 40 rpm for 3 h, washed, and maintained in ND96 at 18 °C. Oocytes were injected with cRNA mixtures (e.g., 32 nL of 500 ng µL^−1^ for *OsCNGC14* and *OsCNGC16*; 500 ng µL^−1^ for *OsNRT1.1B*). For TEVC, currents were recorded 2 days post‐injection using a GeneClamp 200B amplifier (Axon Instruments). Bath solutions contained 20 mm CaCl_2_ + 10 mm KCl and 20 mm CaCl_2_ + 10 mm KNO_3_ adjusted osmotic to 220 mmol kg^−1^ with mannitol. Voltage steps (−140 mV to +20 mV, 10 mV increments) were applied for 3 s. For **^15^** N uptake, oocytes were incubated in **^15^** N‐KNO_3_‐containing solutions (0.2 or 10 mm **^15^** N‐KNO_3_) for 6 h, then washed 5 times, dried at 65 °C for 2 days, and analyzed using an isotope mass spectrometer (DELTA V Advantage, Thermo Fisher).

### In Vivo Degradation Assay

Protoplasts expressing target proteins pre‐cultured in 10 mm KCl or KNO_3_ were treated with 200 µm cycloheximide (CHX) for 0, 45, 90, and 135 min. Pelleted protoplasts were extracted with lysis buffer (25 mm Tris‐HCl (pH 7.5), 1 mm EDTA, 150 mm NaCl, 1% (v/v) Triton X‐100, 5% (v/v) glycerol, and 1x protease and phosphatase inhibitor cocktail). Protein samples were analyzed by SDS‐PAGE and immunoblotting using anti‐FLAG or anti‐Actin antibodies. The X‐ray film was scanned and analyzed for statistics using ImageJ software.

### Cytosol and Nuclear Fractions from Rice Protoplasts

The cytosol and nuclear fractions from plant tissue were separated according to the method described previously.^[^ ^43^ ^]^ After treatment with 10 mm KNO_3_, pelleted protoplasts expressing target proteins were gently resuspended in 200 µL ice‐cold lysis buffer (20 mm Tris‐HCl (pH 7.4), 20 mm KCl, 2.0 mm EDTA, 25% (v/v) glycerol, 2.5 mm MgCl_2_, 250 mm sucrose, 1x protease and phosphatase inhibitor cocktail) and incubated on ice for 10 min. The homogenate was filtered through a 100 and 40 µm nylon mesh sequentially. Lysates were clarified by centrifugation at 1500 × *g* for 20 min at 4 °C, and the supernatant was transferred to a new tube, the nuclei pellet was kept in the tube. The supernatant was further clarified by centrifugation at 20000 × *g* for 20 min at 4 °C and was designated as the cytosol fraction. The nuclei pellet fraction was gently resuspended in 1 mL NRBT buffer (20 mm Tris‐HCl (pH 7.4), 2.5 mm MgCl_2_, 25% (v/v) glycerol, 0.2% (v/v) NP40, 0.1% (v/v) Triton X‐100), and centrifuged at 1500 × *g* for 10 min at 4 °C. The nuclei fraction was washed twice using the NRBT buffer. Finally, the nuclear pellet was resuspended in 100 µL total protein extraction buffer (50 mm Tris‐HCl (pH 7.4), 150 mm NaCl, 1 mm EDTA, 1% (v/v) Triton X‐100, 1x protease and phosphatase inhibitor cocktail), and then eluted by boiling in reducing SDS loading buffer. The cytosolic and nuclear fractions were resolved by SDS‐PAGE and immunoblotted with anti‐FLAG antibody, and anti‐UGPase and anti‐H3 antibodies served as compartment‐specific markers for cytosol and nucleus, respectively. The X‐ray film was scanned and analyzed for statistics using ImageJ software.

### Cytosolic [Ca^2^⁺] Imaging Using Yellow Cameleon

Seeds of *ZH11* ^YC3.6^, *osnrt1.1b* ^YC3.6^, *oscngc14* ^YC3.6^, and *oscngc16* ^YC3.6^ were sterilized and grown on nitrate‐free Kimura medium (0.5 mm (NH_4_)_2_SO_4_, 0.6% agar, pH 5.7) for 12–16 days. Ratiometric Ca^2^⁺ imaging was performed using a Zeiss LSM 710 confocal microscope (excitation: 445 nm; emission: 484–505 nm for CFP, 520–528 nm for FRET).

### Field Cultivation of Rice

To investigate the effects of *oscngc14* and *oscngc16* mutants on N utilization in the field, three large‐scale field trials were conducted from 2022 to 2024 at two experimental stations: the Institute of Genetics and Developmental Biology (IGDB, Beijing) and the IGDB South China Experimental Station (Lingshui, Hainan).

2022 Hainan Trial (2021 December‐April): N source: a mixture of 60% nitrate, 40% ammonium. Low N treatment: 0.5 kg N per 100 m^2^; normal N treatment: 1 kg N per 100 m^2^. Fertilizers: KNO_3_ (nitrate source) and (NH_4_)_2_SO_4_ (ammonium source). Phosphorus fertilizer: P_2_O_5_ (0.5 kg P per 100 m^2^) applied before transplanting. Fertilizer uniformity: N fertilizers were evenly distributed to minimize variability.

2022 Beijing Trial (May‐October): N source: same as above. Low and normal N treatments are identical to Hainan cultivation.

2024 Hainan Trial (2023 December–April): Conditions are identical to the 2022 Hainan and 2022 Beijing cultivation. For large‐scale field tests, the plot size for yield tests was 1.44 m^2^, with each plot containing 36 effective plants. The calculation formula of NUE is as follows: Actual yield per plot (g) / [area of plot (m^2^) x N fertilizer applied (kg)].

To investigate *OsSPX4*‐overexpressing plants grown in rice under field conditions, on field trial was conducted in 2024 at the Institute of Genetics and Developmental Biology (IGDB, Beijing). Low N condition was identical to 2022 Beijing cultivation.

In all field trials, planting spacing: 20 cm. Irrigation and design: Continuous flooding maintained throughout growth; completely randomized block design for each plot. Planting density: 8 rows × 8 plants per plot. Border rows were removed before final analysis to mitigate edge effects.

### Labeling with ^15^N‐Nitrate and ^15^N‐Ammonium for Determining ^15^N Accumulation Assay in Plants

Rice seedlings were grown in a basic nutrient solution with either 0.1 mm (NH_4_)_2_SO_4_ and 0.1 mm KNO_3_ or 1 mm (NH_4_)_2_SO_4_ and 1 mm KNO_3_, with potassium concentrations adjusted, for 14 days. Subsequently, seedlings were transferred to fresh solution supplemented with 0.2/2 mm ^15^N‐NH_4_Cl or 0.1/1 mm ^15^N‐ KNO_3_ (98% ^15^N abundance; Sigma–Aldrich, Cat# 299251 to ^15^N‐NH_4_Cl and Cat#335134 to ^15^N‐KNO_3_) replace 0.1/1 mm (NH_4_)_2_SO_4_ or 0.1/1 mm KNO_3_ for 3 h. After labeling, roots were rinsed with 0.1 mm CaSO_4_ for 1 min, separated from shoots, ground into powder, and analyzed for **^15^** N content using an isotope ratio mass spectrometer (Thermo Finnigan Delta Plus XP coupled with a Flash EA 1112 elemental analyzer). Each biological replicate included 20 seedlings (*n* = 4 biological replicates).

### Measurement of Nitrogen Concentration

Rice seedlings were grown in 0.1 mm (NH_4_)_2_SO_4_ and 0.1 mm KNO_3_ or 1 mm (NH_4_)_2_SO_4_ and 1 mm KNO_3_ in basic nutrient solution for 14 days. The total N concentration was determined by the Kjeldahl method as described.^[^ ^44^ ^]^ Each biological replicate included 20 seedlings (*n* = 4 biological replicates).

### Phosphorylation Assay in Rice Protoplast

To examine OsNLP3 phosphorylation under nitrate induction, nitrate‐free protoplasts stably expressing OsNLP3‐FLAG were treated with W5 solution containing 10 mm KNO_3_ for 15 or 30 min, with or without 2 mm LaCl_3_ pretreatment for 10 min. To test the effect of Ser193 on OsNLP3 phosphorylation, nitrate‐free protoplasts transfected with p*CAMBIA2300‐35S‐OsNLP3‐FLAG*, p*CAMBIA2300‐35S‐OsNLP3^S193A^‐FLAG*, and p*CAMBIA2300‐35S‐OsNLP3^S193D^‐FLAG* were incubated in W5 solution for 12 h, centrifuged at 200 × *g*, and treated with 10 mm KNO_3_ for 30 min. To investigate the effects of OsNRT1.1B, OsCNGC14, and OsCNGC16 on OsNLP3 phosphorylation, nitrate‐free protoplasts of *osnrt1.1b*, *oscngc14*, and *oscngc16* mutants transfected with p*CAMBIA2300‐35S‐OsNLP3‐FLAG* were incubated in W5 solution for 12 h, centrifuged at 200 × *g*, and treated with 10 mm KNO_3_ for 30 min. Total proteins were extracted from lysed protoplasts using 50 µL extraction buffer (50 mm HEPES (pH 7.5), 150 mm NaCl, 1% Triton X‐100, 10% glycerol, 1× Protease and phosphatase inhibitor cocktail (NCM Biotech) at 4 °C for 30 min. Then treatment with or without λPP (Lambda Protein Phosphatase) (NEB) at 30 °C for 30 min, mixed with 2× SDS loading buffer at 100 °C for 10 min, and analyzed by SDS‐PAGE and Phos‐tag gels, immunoblotting using HRP‐conjugated anti‐FLAG. The X‐ray film was scanned and analyzed for statistics using ImageJ software.

### Dual‐Luciferase Reporter Assay

Dual‐luciferase reporter assay was performed using nitrate‐free rice protoplasts. Protoplasts were isolated from 12‐day‐old seedlings and transformed as previously described.^[^ ^42^ ^]^ The *pGreenII‐0800‐pNRT2.1‐LUC*, *pGreenII‐0800‐pNIA1‐LUC*, *pGreenII‐0800‐pNIR‐LUC*, *pGreenII‐0800‐pGS1.1‐LUC*, and *pGreenII‐0800‐pNLP3‐LUC* vectors served as the reporters. The *pCAMBIA2300*‐*35S‐OsNLP3‐FLAG, pCAMBIA2300*‐*35S‐OsNLP3^S193A^‐FLAG*, and *pCAMBIA2300*‐*35S‐OsNLP3^S193D^‐FLAG* constructs described above were used as the effector. The empty *pCAMBIA2300‐35S ‐FLAG* vector served as the control. Protoplasts co‐expressing effector and reporters were incubated in W5 solution for 12 h. Then, 5 mm KNO_3_ was used as a treatment, and 5 mm KCl was added as control for another 0–180 min of incubation. Firefly luciferase (LUC) and Renilla luciferase (REN) activities were measured using a Dual Luciferase reporter assay kit (Promega, E1960) with a GloMax 20/20 luminometer (Promega). LUC/REN ratios were calculated to represent the relative LUC activity. For each plasmid combination, three independent transformations were performed.

### Nitrate Induction Assay of Nitrate‐Responsive Genes by Different OsNLP3 Phospho‐Forms

Nitrate‐free *osnlp3* protoplasts expressed with *pCAMBIA2300*‐*35S‐OsNLP3‐FLAG, pCAMBIA2300*‐*35S‐OsNLP3^S193A^‐FLAG*, and *pCAMBIA2300*‐*35S‐OsNLP3^S193D^‐FLAG* were incubated in W5 solution for 12 h. Then, 5 mm KNO_3_ was added to the protoplasts as treatment, and 5 mm KCl was added as a control, followed by incubation for another 0–90 min. Protoplasts were collected by centrifuging at 200 × *g* for 3 min for gene expression analysis.

### Statistical Analysis

All experiments were independently repeated in at least three biological replicates. Data are presented as mean ± SD. Statistical analyses were performed using GraphPad Prism 10.4 software. For comparisons between two groups with normally distributed data, Student's *t*‐test was used. For comparisons among three or more groups, one‐way ANOVA followed by Tukey's test was employed. Statistical significance was defined as *P* < 0.05. Detailed statistical methods for each experiment are provided in the corresponding figure legends.

## Conflict of Interest

The authors declare no conflict of interest.

## Author Contributions

X.W., Y.L., and W.L. contributed equally to this work. X.W., designed research, performed experiments, analyzed the data, and wrote the manuscript. W.L., Y.L., X.M., W.W., Z.J., and Y.W., conducted some of the experiments. Y.L., L.L., B.H., and C.C. designed research, wrote the manuscript and supervised the project.

## Supporting information



Supporting Information

## Data Availability

The data that support the findings of this study are available from the corresponding author upon reasonable request.
