# Supplementary material for: OsNRT1.1B‐OsCNGC14/16‐Ca2+‐OsNLP3 Pathway: Phosphorylation‐Mediated Maintenance of Nitrogen Homeostasis
Source: Adv Sci (Weinh). 2025 Sep 3;12(43):e07919. doi: 10.1002/advs.202507919 (PMC12631913; doi:10.1002/advs.202507919)
Supplement: Supplementary file 1 — Supporting Information [file ADVS-12-e07919-s001.docx]

**Supplemental Data**

**
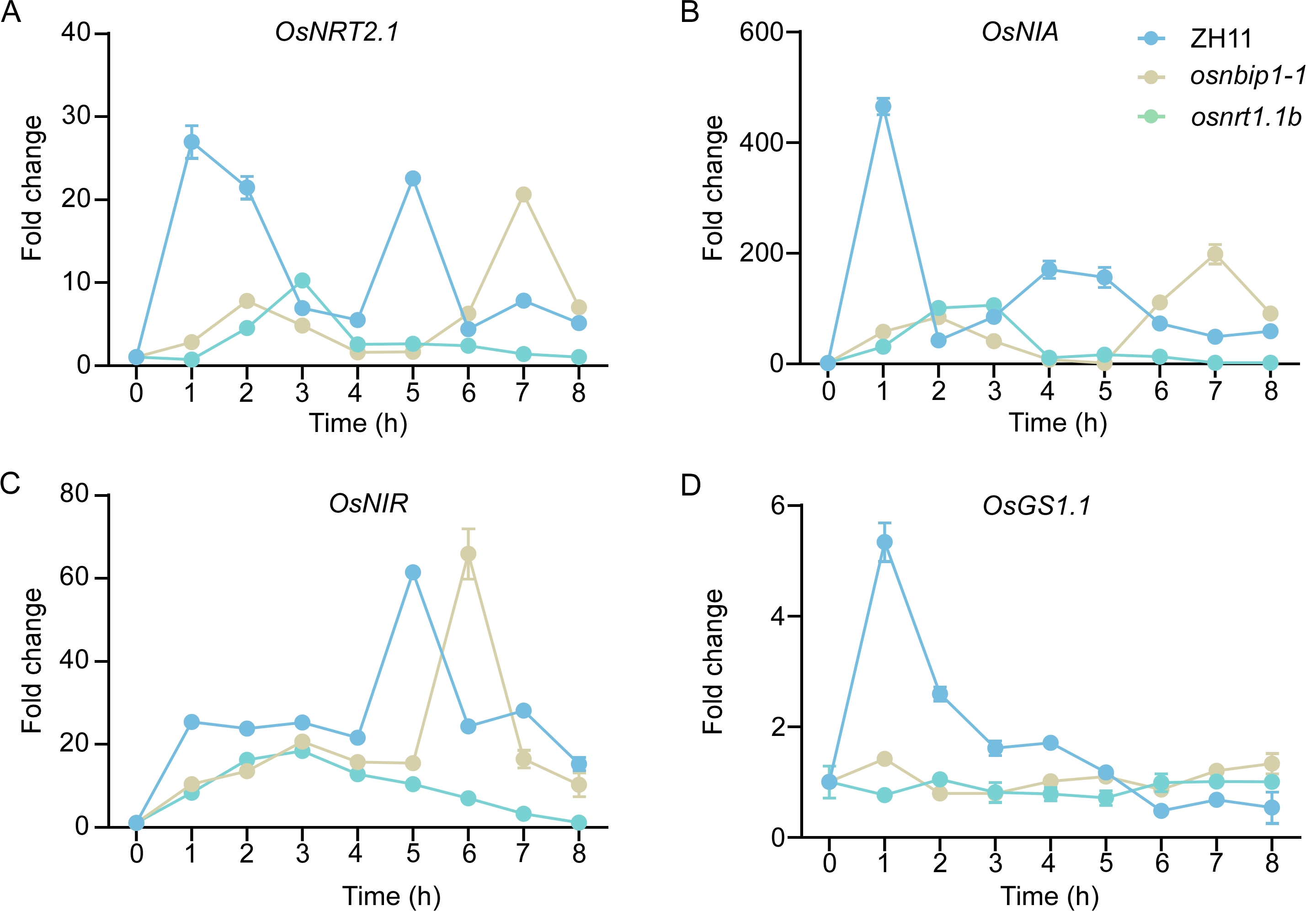
**

**Supplemental Figure 1. The** **induced expression of nitrogen-related genes exhibits a biphasic pattern.**

RT-qPCR analysis of N-related genes in roots of ZH11, *osnbip1-1*, and *osnrt1.1b* plants treated with 5 mM KNO_3_ or KCl (control) for 0-8 hours. Relative expression levels of **(A)** *OsNRT2.1*, **(B)** *OsNIA*, **(C)** *OsNIR*, and **(D)** *OsGS1.1* were normalized to KCl-treated controls (set as 1). Means ± SD. (*n = 3* biological replicates).

**
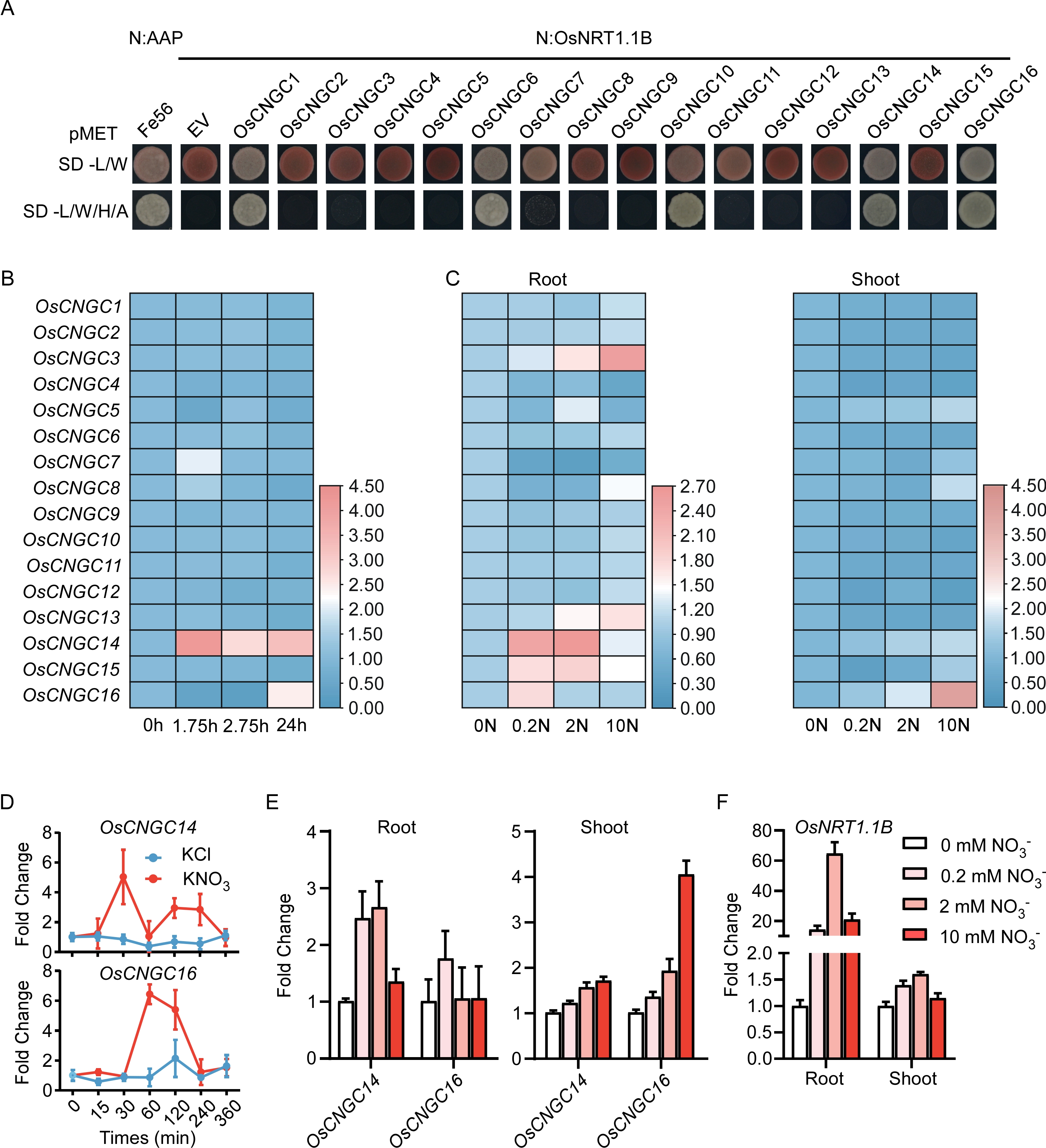
**

**Supplemental Figure 2.** **OsCNGC14 and OsCNGC16 are prime candidates for mediating nitrate-induced Ca²⁺ influx.**

**A.** OsNRT1.1B interacts with OsCNGCs in a split-ubiquitin yeast two-hybrid assay. Membrane protein interactions were tested using the split-ubiquitin system. SD medium lacking Leu and Trp assay (SD -L/W) (top); SD medium lacking Leu, Trp, His, Ade, and with 5 mM 3-AT assay (SD -L/W/H/A) (bottom). N: APP/pMET: Fe65 was used as a positive control; N: OsNRT1.1B/pMET: EV was used as a negative control.

**B.** Nitrate induction of *OsCNGC* expression. Heatmap clustering of *OsCNGC* transcription in ZH11 roots treated with 5 mM KNO_3_ for 0, 1.75, 2.75, and 24 hours. Expression levels were normalized to 0-hour controls (set as 1).

**C.** Dose-effect of *OsCNGC* expression under increasing nitrate supply. Heatmap clustering of *OsCNGC* transcripts in ZH11 roots (left) and shoots (right) exposed to 0-10 mM KNO_3_ for 14 days. Data were normalized to 0 mM nitrate hydroponic culture controls (set as 1).

**D.** Nitrate induction of *OsCNGC14* and *OsCNGC16* expression analysis. Time-course RT-qPCR of *OsCNGC14* and *OsCNGC16* in ZH11 roots after 0-360 minutes of 5 mM KNO_3_ treatment. Data were normalized to the 0-minute control (set as 1). Means ± SD. (*n* = 3 biological replicates).

**E-F.** RT-qPCR assay of ZH11 under rising KNO_3_ hydroponic culture for 14 days. **E.** Tissue-specific *OsCNGC14* and *OsCNGC16* response to nitrate availability. *OsCNGC14* and *OsCNGC16* expression in roots (left) and shoots (right) under increasing KNO_3_ concentrations (0-10 mM). **F.** Tissue-specific *OsNRT1.1B* response to nitrate availability. *OsNRT1.1B* expression in roots and shoots with raising KNO_3_ concentrations (0-10 mM). Data normalized to 0 mM nitrate hydroponic culture control (set as 1). Means ± SD. (*n* = 3 biological replicates).


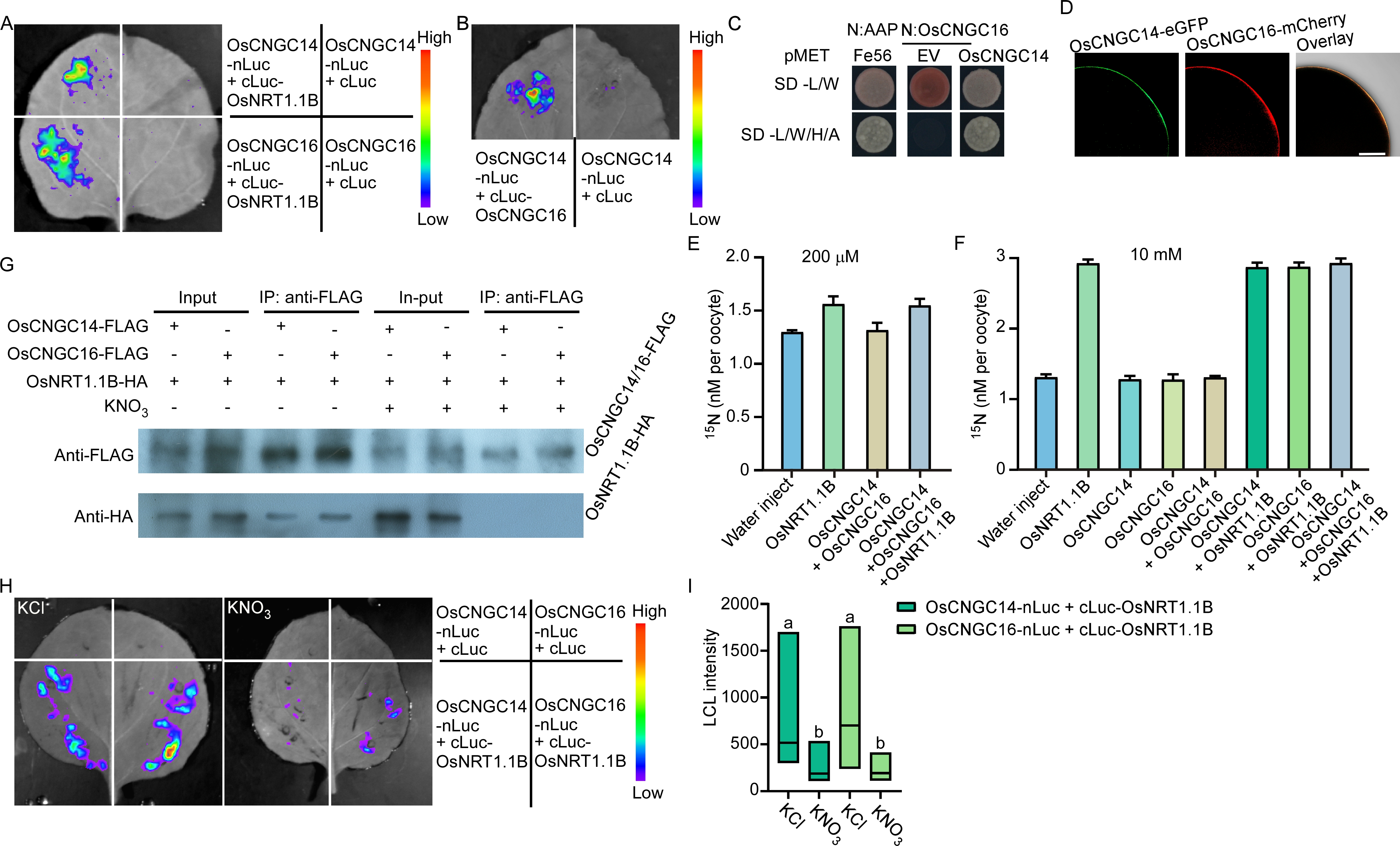


**Supplemental Figure 3. The OsNRT1.1B-OsCNGC14/16 interaction does not affect OsNRT1.1B transport function but is dynamically regulated by nitrate.**

**A.** OsCNGC14 and OsCNGC16 directly interact with OsNRT1.1B *in planta*. Luminescence complementation imaging (LCI) assay in *N. benthamiana* leaves co-expressing OsCNGC14-nLUC + cLUC-OsNRT1.1B and OsCNGC16-nLUC + cLUC-OsNRT1.1B.

**B.** OsCNGC14 directly interacts with OsCNGC16 in *planta*. LCI assay in *N. benthamiana* leaves co-expressing OsCNGC14-nLUC + cLUC-OsCNGC16.

**C.** OsCNGC14/16 heterocomplex formation in yeast membranes. Membrane protein interactions were tested using the split-ubiquitin system. SD medium lacking Leu and Trp assay (SD -L/W) (top); SD medium lacking Leu, Trp, His, Ade, and with 5 mM 3-AT assay (SD -L/W/H/A) (bottom). N: APP/pMET: Fe65 was used as a positive control; N: OsCNGC16/pMET: EV was used as a negative control.

**D.** Coordinated membrane localization of OsCNGC14 and OsCNGC16 in *Xenopus* oocytes. Confocal images of oocytes co-expressing *OsCNGC14-eGFP and* *OsCNGC16-mCherry* are shown. Left, GFP (488 nm excitation), middle, mCherry (561 nm excitation), and right, overlay (GFP, mCherry, and DIC) of the same sample. Scale bar = 200 μm.

**E-F.** Functional decoupling of OsCNGC14/16 from nitrate transport**.** **E.** Nitrate uptake assay in *Xenopus* oocytes expressing *OsNRT1.1B, OsCNGC14 + OsCNGC16,* and *OsCNGC14 + OsCNGC16+OsNRT1.1B* in 200 μM ^15^N-nitrate. Means ± SD. (*n* = 8 oocytes). **F.** Nitrate uptake assay in *Xenopus* oocytes expressing with *OsNRT1.1B, OsCNGC14, OsCNGC16, OsCNGC14 + OsCNGC16, OsCNGC14 + OsNRT1.1B, OsCNGC16 + OsNRT1.1B,* and *OsCNGC14 + OsCNGC16 + OsNRT1.1B* in 10 mM ^15^N-nitrate. Means ± SD. (*n* = 8 oocytes).

**G.** Nitrate-responsive modulation of OsCNGC14 or OsCNGC16 and OsNRT1.1B interaction. Co-immunoprecipitation (Co-IP) assay in ZH11 protoplasts co-expressing OsCNGC14-FLAG + OsNRT1.1B-HA or OsCNGC16-FLAG + OsNRT1.1B-HA*,* treated with 10 mM KNO_3_ or KCl for 30 minutes.

**H**. The nitrate effect on the interaction between OsCNGC14 or OsCNGC16 and OsNRT1.1B. LCI assay in *N. benthamiana* leaves co-expressing OsCNGC14-nLUC + cLUC-OsNRT1.1B and OsCNGC16-nLUC + cLUC-OsNRT1.1B in the absence or presence of nitrate (10 mM KCl or KNO_3_ for 30 minutes).

**I**. Quantitative analysis of the nitrate effect on interaction between OsCNGC14 or OsCNGC16 and OsNRT1.1B. Means ± SD. (*n* = 7 leaves; one-way ANOVA with Tukey’s multiple comparisons test, *P < 0.05*).

**
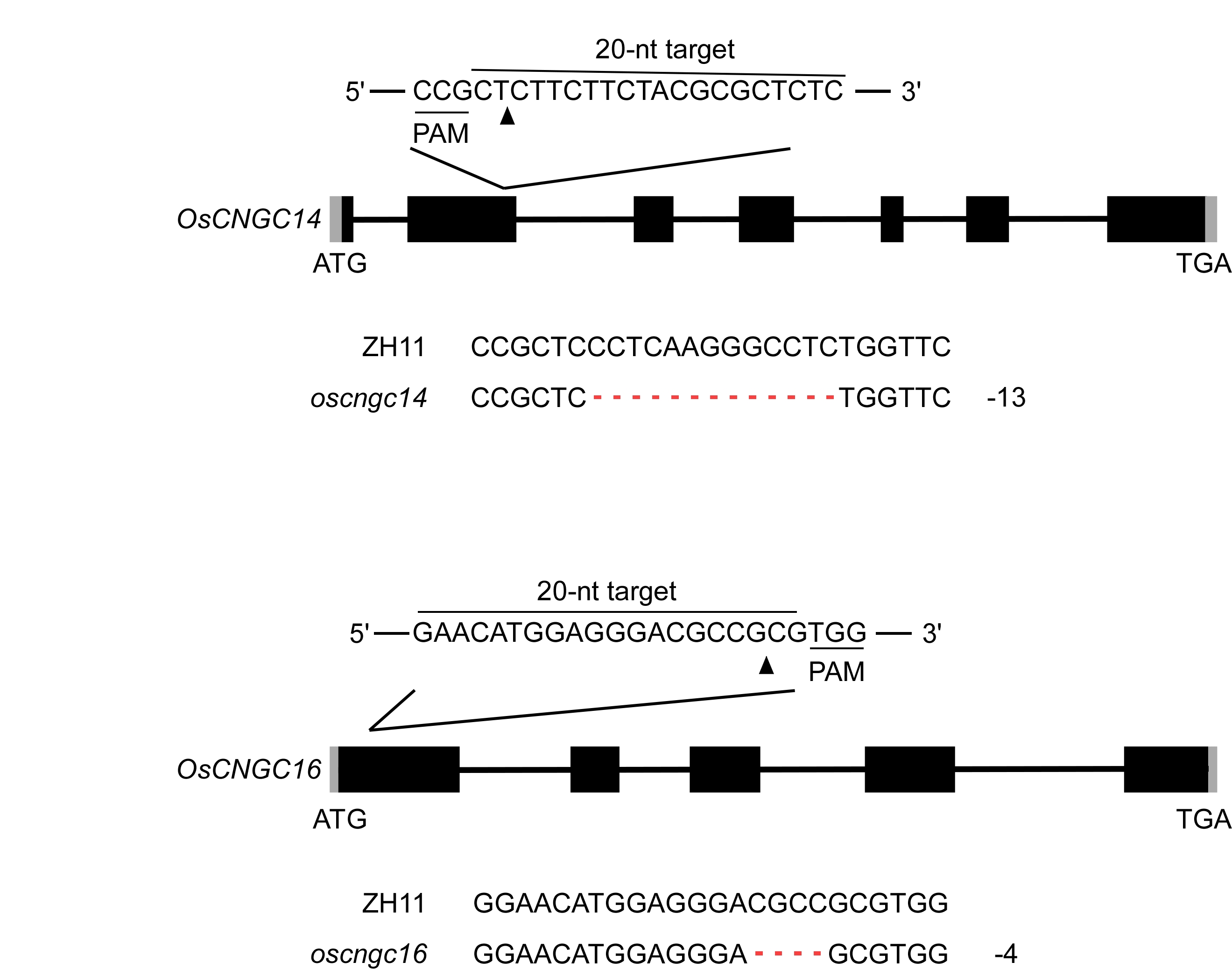
**

**Supplemental Figure 4. *oscngc14* and *oscngc16* were generated by *CRISPR/Cas9* editing.**

The 20-nt *OsCNGC14* and *OsCNGC16* target sequences are located upstream of the protospacer adjacent motif (PAM), with Cas9 cut sites indicated within the first or second intron. Sequencing analysis of *OsCNGC14* and *OsCNGC16* gene in the CRISPR/Cas9-generated os*cngc14* and *oscngc16* mutants were performed. PCR fragments corresponding to Os*CNGC14* in os*cngc14* and Os*CNGC16* in os*cngc16* were amplified, sequenced, and aligned to ZH11 coding sequences. The minus (-) sign indicates the number of nucleotides. The reverse complement of the PAM sequence is underlined in black, and black arrowheads indicate the theoretical Cas9 cleavage sites.

**
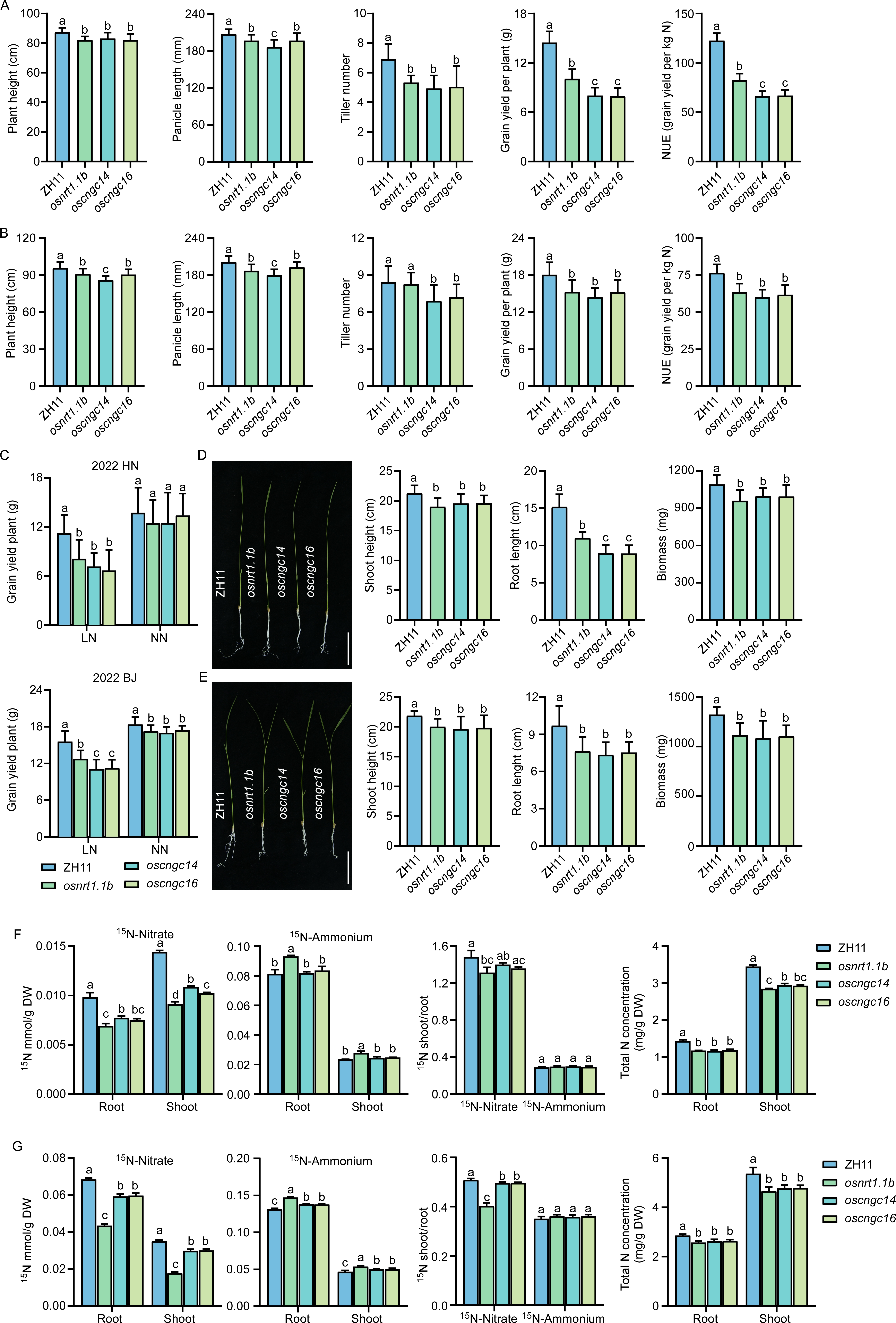
**

**Supplemental Figure 5.** **The *oscngc14* and *oscngc16* mutants display severe growth defects, impaired nitrogen utilization, and reduced grain yield.**

**A-B:** Agronomic performance of *osnrt1.1b,* *oscngc14,* and *oscngc16* mutants under different N regimes. **A.** Low N field. **B.** Normal N field. Field trials in Hainan (2024) comparing ZH11, *osnrt1.1b*, *oscngc14*, and *oscngc16* plants under different N regimes. Plant height, panicle length, tiller number per plant, grain yield per plant, and NUE were measured. Means ± SD. (*n* = 18 plants, 6 replicates for actual yield per plot and NUE, one-way ANOVA with Tukey’s multiple comparisons test, *P < 0.05*).

**C.** Geographic consistency of yield penalties in *osnrt1.1b,* *oscngc14*, and *oscngc16* mutants. Grain yield per plant of ZH11 and mutants under low N and normal N conditions in Hainan (2022) and Beijing (2022). Means ± SD. (*n* = 18 plants, one-way ANOVA with Tukey’s multiple comparisons test, *P < 0.05*).

**D** and **E.** Low and normal N cultivation led to growth defects in *osnrt1.1b,* *oscngc14*, and *oscngc16* mutants. ZH11, *osnrt1.1b*, *oscngc14*, and *oscngc16* plants were grown in hydroponic solution with **D**. 0.1 mM nitrate and 0.2 mM ammonium or **E**. 1 mM nitrate and 2 mM ammonium for 14 days. The representative photographs were captured (scale bars, 5 cm), shoot height, root length, and biomass were measured and quantified. Means ± SD. (*n = 18* plants) (one-way ANOVA with Tukey’s multiple comparisons test, *P < 0.05*).

**F** and **G.** Disrupted N allocation and homeostasis in *osnrt1.1b,* *oscngc14*, and *oscngc16* mutants. ZH11, *osnrt1.1b*, *oscngc14*, and *oscngc16* plants were grown in hydroponic solution with **F**. 0.1 mM nitrate and 0.2 mM ammonium or **G**. 1 mM nitrate and 2 mM ammonium for 14 days. ^15^N accumulation in roots and shoots of ZH11, *osnrt1.1b*, *oscngc14*, and *oscngc16* plants labeled with ^15^N-nitrate or ^15^N-ammonium, root-to-shoot transport of nitrate or ammonium, and total N concentrations were measured and quantified. Means ± SD. (*n = 4* biological replicates) (one-way ANOVA with Tukey’s multiple comparisons test, *P < 0.05*).

**
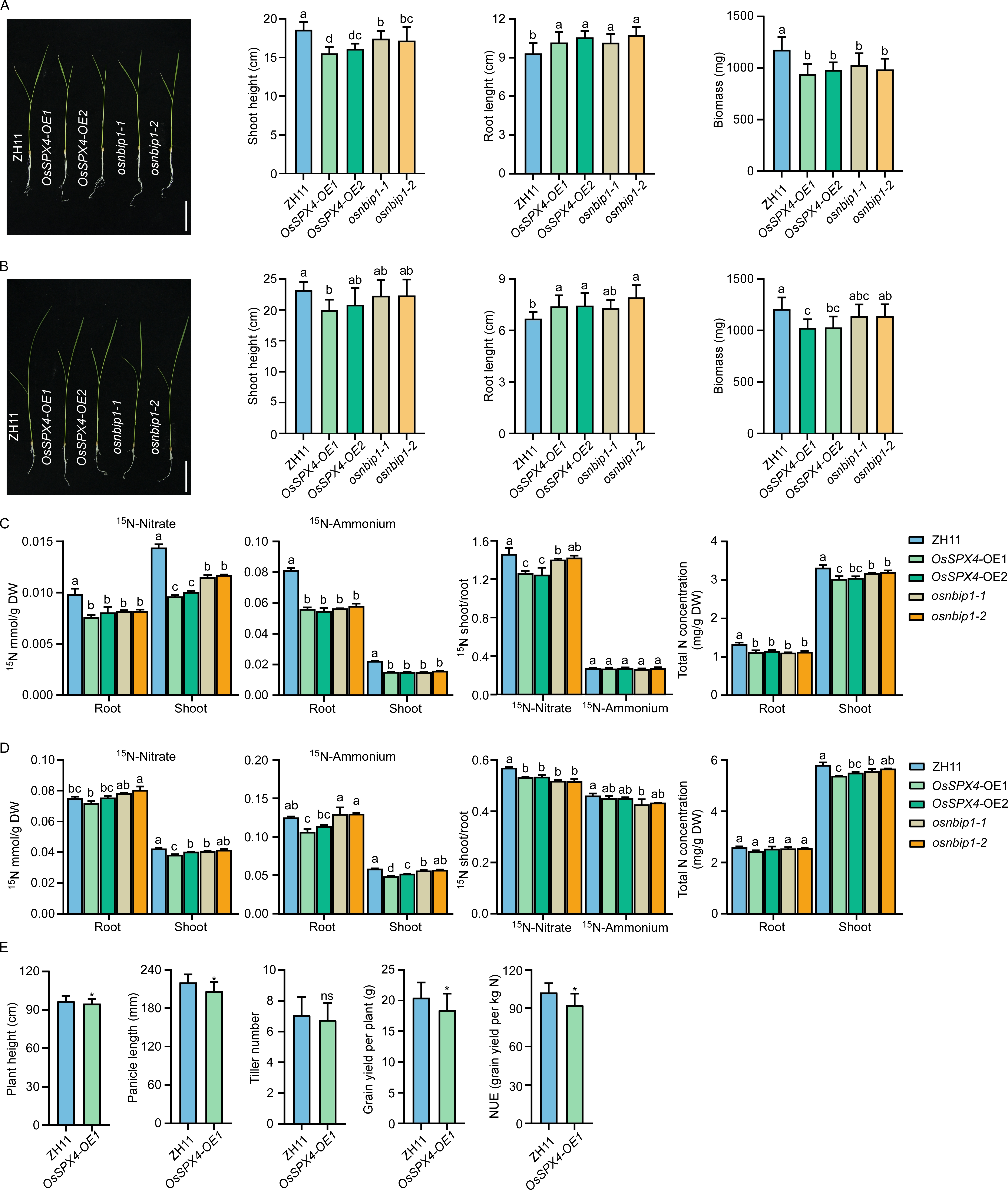
**

**Supplemental Figure 6. Growth and nitrogen utilization are slightly suppressed in *OsSPX4*-overexpressing plants and *osnbip1* mutants.**

**A** and **B.** Low and normal N levels caused growth defects in *OsSPX4-*OE*,* and *osnbip1* plants. ZH11, *OsSPX4-*OE1/2*,* and *osnbip1-1*/*2* plants were grown in hydroponic solutions with **A**. 0.1 mM nitrate and 0.2 mM ammonium or **B**. 1 mM nitrate and 2 mM ammonium for 14 days. The representative photographs were captured. Scale bars, 5 cm. Shoot height, root length, and biomass were measured and quantified. Means ± SD. (*n = 12* plants) (one-way ANOVA with Tukey’s multiple comparisons test, *P < 0.05*).

**C** and **D.** Disrupted N allocation and homeostasis in *OsSPX4-*OE*,* and *osnbip1* plants. ZH11, *OsSPX4-*OE1/2*,* and *osnbip1-1*/*2* plants were grown in hydroponic solutions with **C**. 0.1 mM nitrate and 0.2 mM ammonium or **D**. 1 mM nitrate and 2 mM ammonium for 14 days. ^15^N accumulation assays in roots and shoots of ZH11, *OsSPX4-*OE1/2*,* and *osnbip1-1*/*2* plants labeled with ^15^N-nitrate or ^15^N-ammonium, N root-to-shoot transport assay, and total N concentrations were measured and quantified. Means ± SD. (*n = 4* biological replicates) (one-way ANOVA with Tukey’s multiple comparisons test, *P < 0.05*).

**E**. Agronomic traits of WT (ZH11) and *OsSPX4-*OE1 grown in the field under low N conditions (Beijing, 2024). Plant height, panicle length, tiller number per plant, grain yield per plant, and NUE were measured. Means ± SD (*n* = 18 plants, 6 replicates for actual yield per plot and NUE, two-tailed with Student *t*-test, * *P < 0.05*)**.**


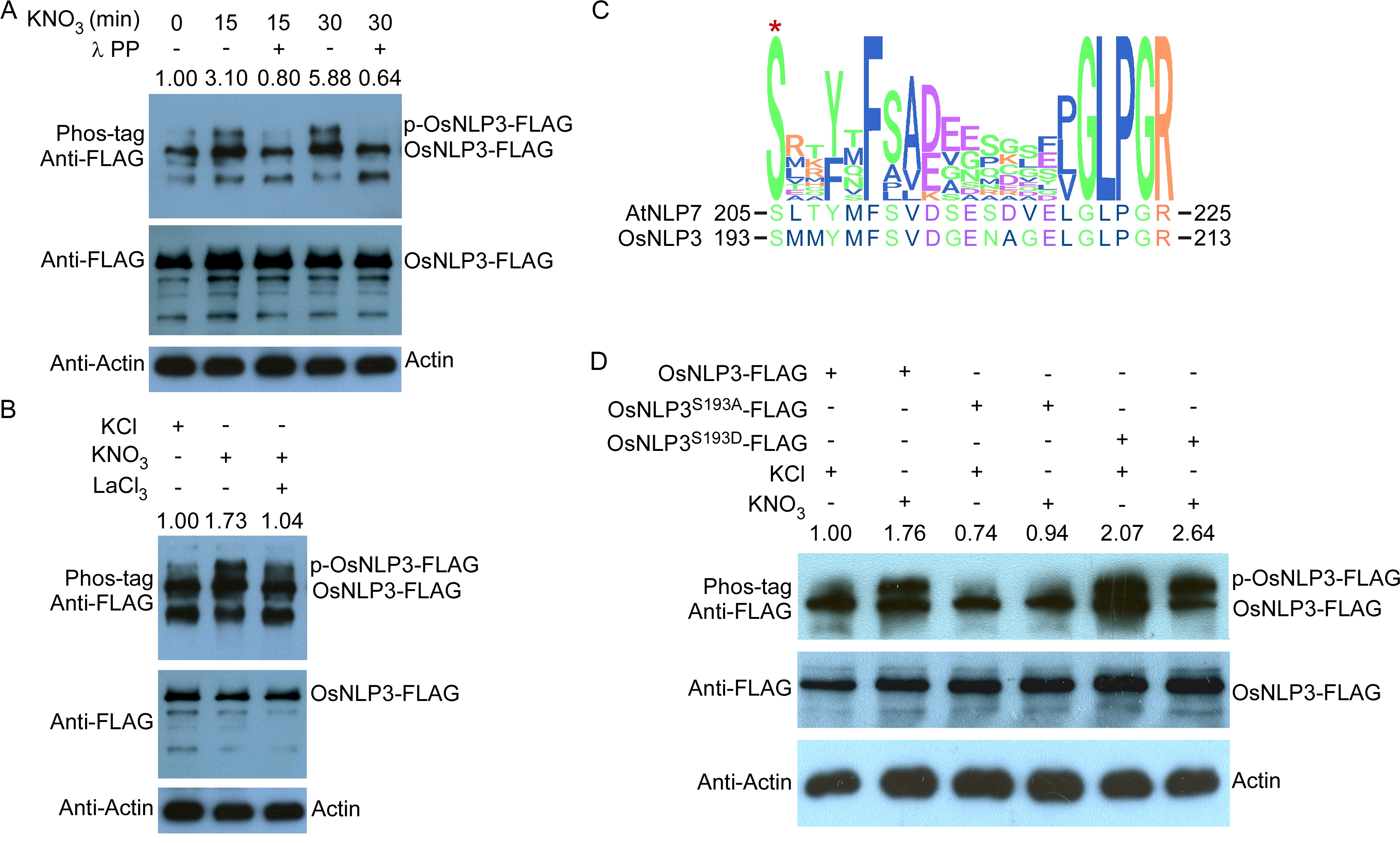


**Supplemental Figure 7. Nitrate-induced OsNLP3 phosphorylation is dependent on Ca^2+^.**

**A.** Time-course phosphorylation of OsNLP3-FLAG in response to nitrate. Phos-tag and SDS-PAGE analysis in protoplasts from *OsNLP3-FLAG-*OE plants, treated with 10 mM KNO_3_ for 15 or 30 minutes. λPP (Lambda Protein Phosphatase) treatment confirms phosphorylation-dependent mobility shift. Phosphorylation intensity (normalized to 0-minute control, set as 1) was quantified from 8% Phos-tag gels (top). Total protein levels verified by 10% SDS-PAGE (bottom). Actin serves as loading control.

**B.** Calcium influx is required for nitrate-induced OsNLP3 phosphorylation. Phos-tag analysis of OsNLP3-FLAG phosphorylation in protoplasts from *OsNLP3-FLAG-*OE plants, pre-treated with 2 mM LaCl₃, followed by treatment with 10 mM KNO_3_. Phosphorylation intensity (normalized to KCl-treated controls, set as 1) was quantified from 8% Phos-tag gels (top). Total protein levels verified by 10% SDS-PAGE (middle). Actin serves as loading control (bottom).

**C.** Conserved phosphorylation residue within the GAF domain of 9 AtNLPs and 5 OsNLPs involving the conserved phospho-residues (marked with a red star). Snap-shot of protein sequence alignments with the GAF motif of OsNLP3 and AtNLP7.

**D.** S193A and S193D mutations alter OsNLP3 phosphorylation dynamics. Phos-tag analysis of OsNLP3-FLAG, OsNLP3^S193A^-FLAG, and OsNLP3^S193D^-FLAG in protoplasts from ZH11, treated with 10 mM KNO_3_ or KCl for 30 minutes. Phosphorylation intensity (normalized to KCl-treated OsNLP3-FLAG, set as 1) was quantified from 8% Phos-tag gels (top). Total protein levels verified by 10% SDS-PAGE (middle). Actin serves as loading control (bottom).


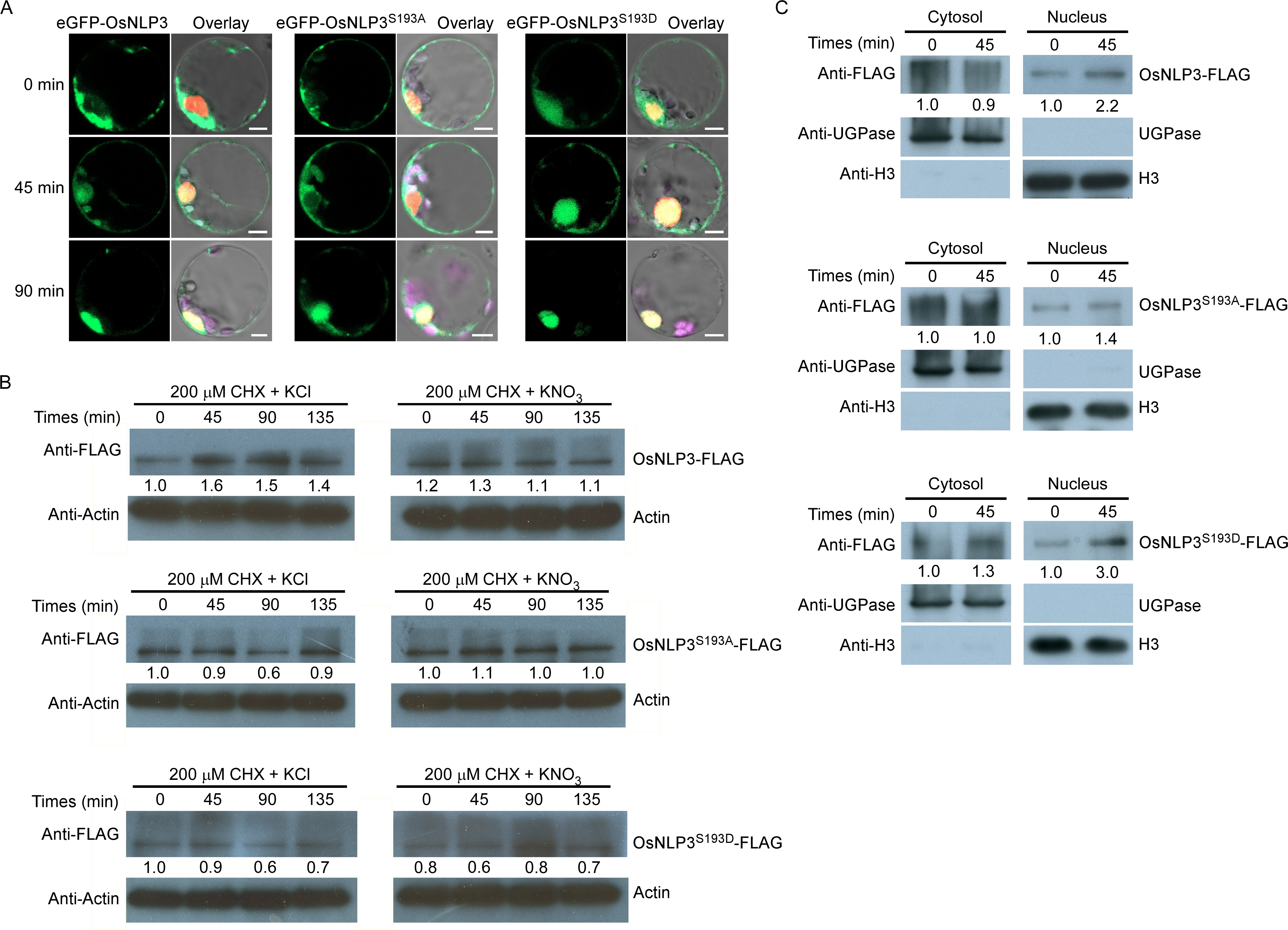


**Supplemental Figure 8.** **Nitrate induces phosphorylation of OsNLP3 and accelerates its nuclear translocation.**

**A.** Phosphorylation at Ser193 accelerates OsNLP3 nuclear translocation in response to nitrate. Confocal images of eGFP-OsNLP3, eGFP-OsNLP3^S193A^, and eGFP-OsNLP3^S193D^ in *osnlp3* cells treated with 5 mM KNO_3_ or KCl, respectively. Left, GFP (488 nm excitation). Right, overlay (GFP, mCherry, and DIC) from the same sample. Images captured at 0, 45, and 90 minutes after treatment. Scale bars = 5 µm.

**B.** Nitrate treatment does not affect the protein stability of OsNLP3, OsNLP3^S193A^ or OsNLP3^S193D^. ZH11 protoplasts expressing OsNLP3-FLAG, OsNLP3^S193A^-FLAG, and OsNLP3^S193D^-FLAG were treated with 10 mM KCl or KNO_3_ and 200 µM CHX for the specified duration. Protein abundance was detected by anti-FLAG immunoblotting (top). Actin serves as loading control (normalized to 0-minute KCl treatment, set as 1) (bottom).

**C**. Nucleus and cytoplasm fractionation assays demonstrated that phosphorylation accelerates OsNLP3 nuclear translocation under nitrate treatment. Subcellular localization immunoblotting analysis of different OsNLP3 phospho-forms (wild-type, S193D, and S193A) following 45-minute treatment with 10 mM KNO_3_. Protein abundance was detected by anti-FLAG immunoblotting (top). UGPase (middle) and H3 (bottom) were used as the cytoplasm and nucleus controls, respectively (normalized to 0-minute, set as 1).


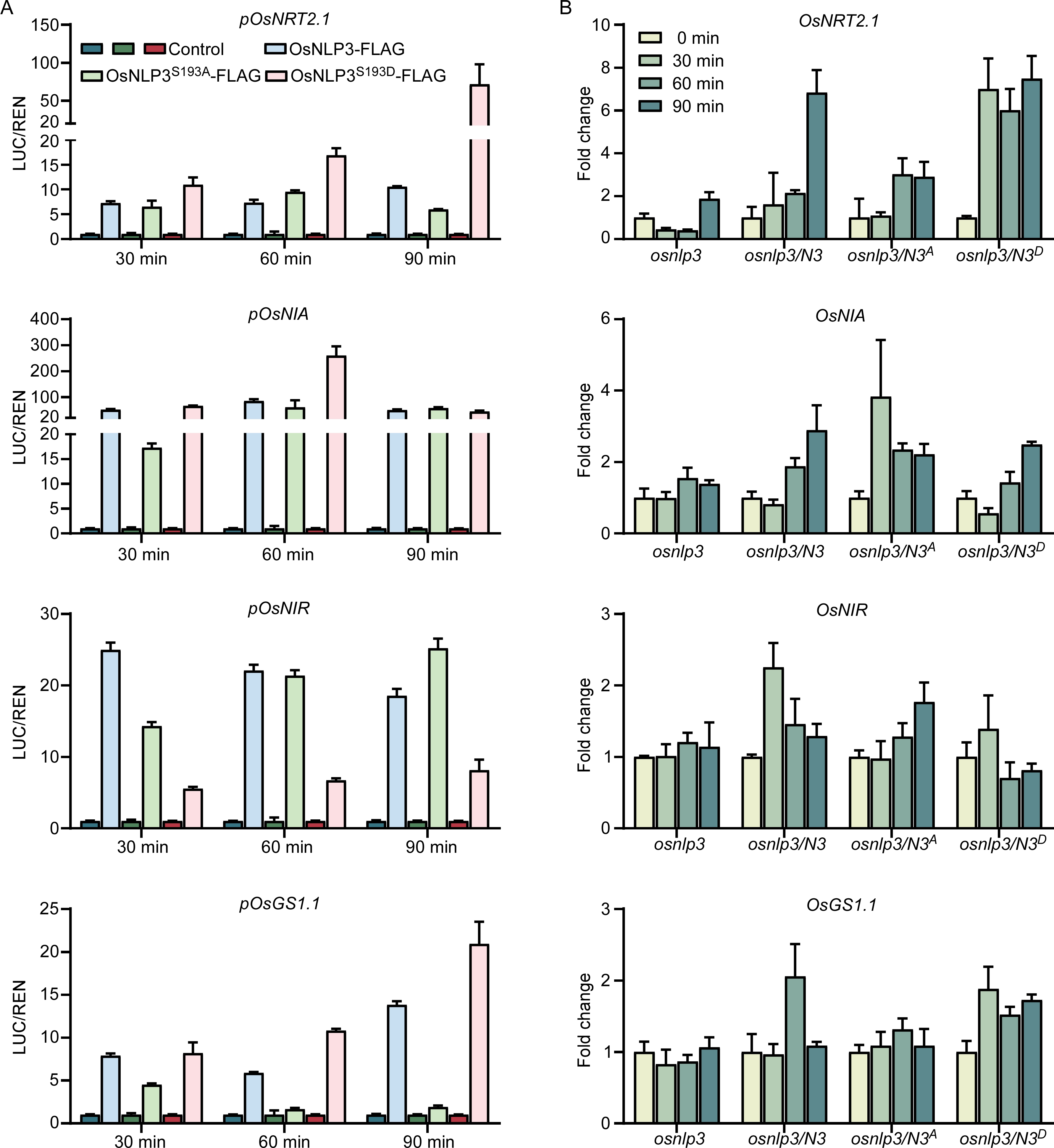


**Supplemental Figure 9. OsNLP3 phosphorylation potentiates its transcriptional activity on nitrogen-responsive genes under nitrate treatment.**

**A.** Phosphorylation at Ser193 changes *OsNLP3* transcriptional activation. Transcriptional activity assays of OsNLP3-FLAG, OsNLP3^S193A^-FLAG, or OsNLP3^S193D^-FLAG with the promoters of *OsNRT2.1*, *OsNIA*, *OsNIR*, and *OsGS1.1* were performed. The dual-luciferase reporter plasmids driven by each promoter were transiently expressed in *osnlp3* protoplasts together with empty vector (control) or OsNLP3 effectors (OsNLP3-FLAG, OsNLP3^S193A^-FLAG, or OsNLP3^S193D^-FLAG), respectively. Luciferase activity was measured at 30, 60, and 90 minutes after 5 mM KNO_3_ treatment. Data are normalized to *OsNLP3*-FLAG control (set as 1). Mean ± SD. (*n = 3* biological replicates).

**B.** Time-course induction of nitrate-responsive genes by different OsNLP3 phospho-forms. RT-qPCR analysis measured expression levels of *OsNRT2.1*, *OsNIA*, *OsNIR*, and *OsGS1.1* in *osnlp3* protoplasts expressing *OsNLP3-FLAG* (*N3*), *OsNLP3^S193A^-FLAG* (*N3^A^*), or *OsNLP3^S193D^-FLAG* (*N3^D^*), respectively. The expression levels were measured at 0, 30, 60, and 90 minutes after 5 mM KNO_3_ or KCl (control) treatment. Data are normalized to 0-minute controls (set as 1). Mean ± SD. (*n = 3* biological replicates).


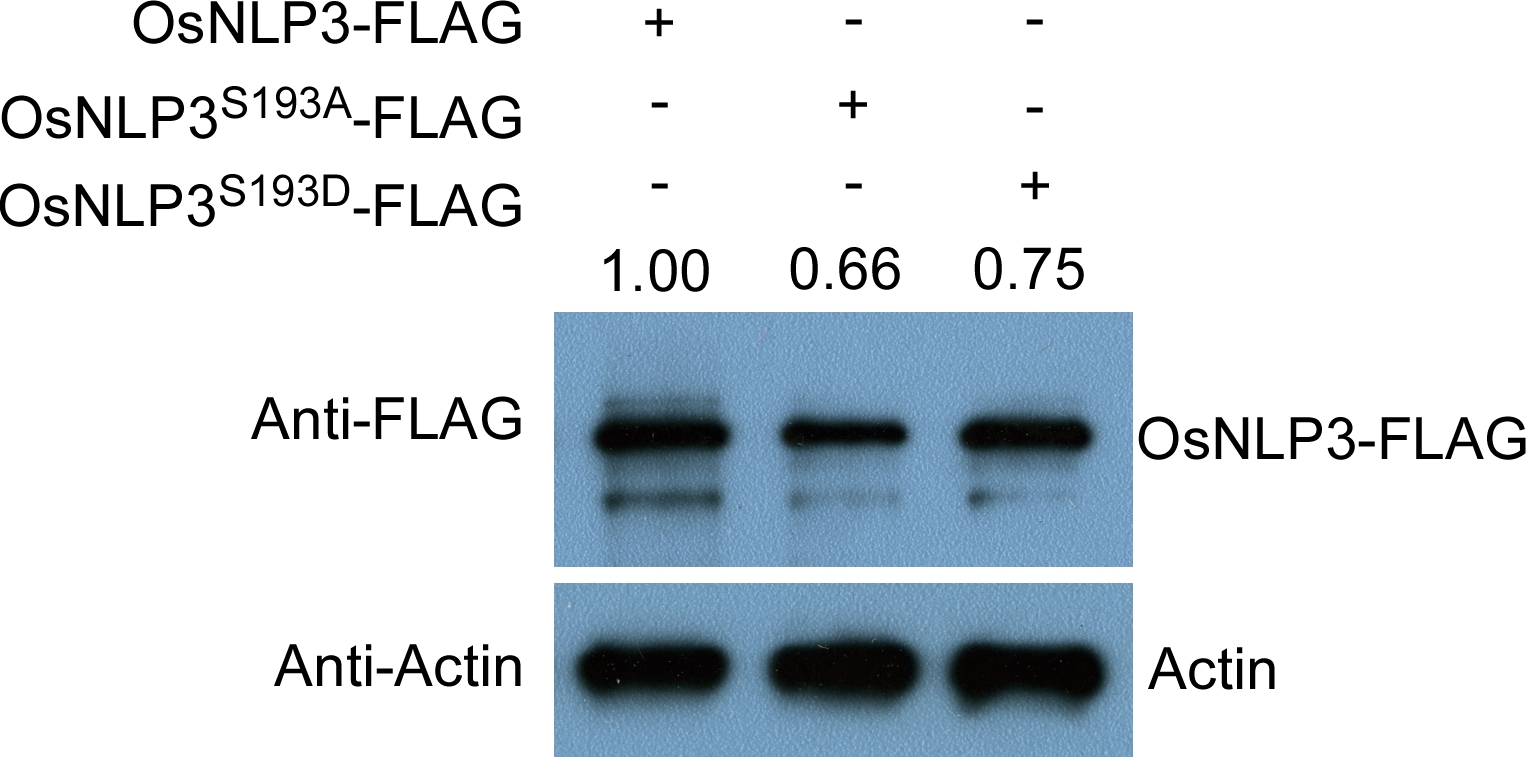


**Supplemental Figure 10.** **The protein levels of OsNLP3-FLAG, OsNLP3^S193A^-FLAG or OsNLP3^S193D^-FLAG in *osnlp3* protoplasts.**

Total proteins were extracted from *osnlp3* protoplasts expressing Os*NLP3-FLAG*, *OsNLP3^S193A^-FLAG* or *OsNLP3^S193D^-FLAG*. OsNLP3 protein levels were quantified from 10% SDS-PAGE (normalized to OsNLP3-FLAG controls, set as 1) (top). Actin serves as a loading control (bottom).


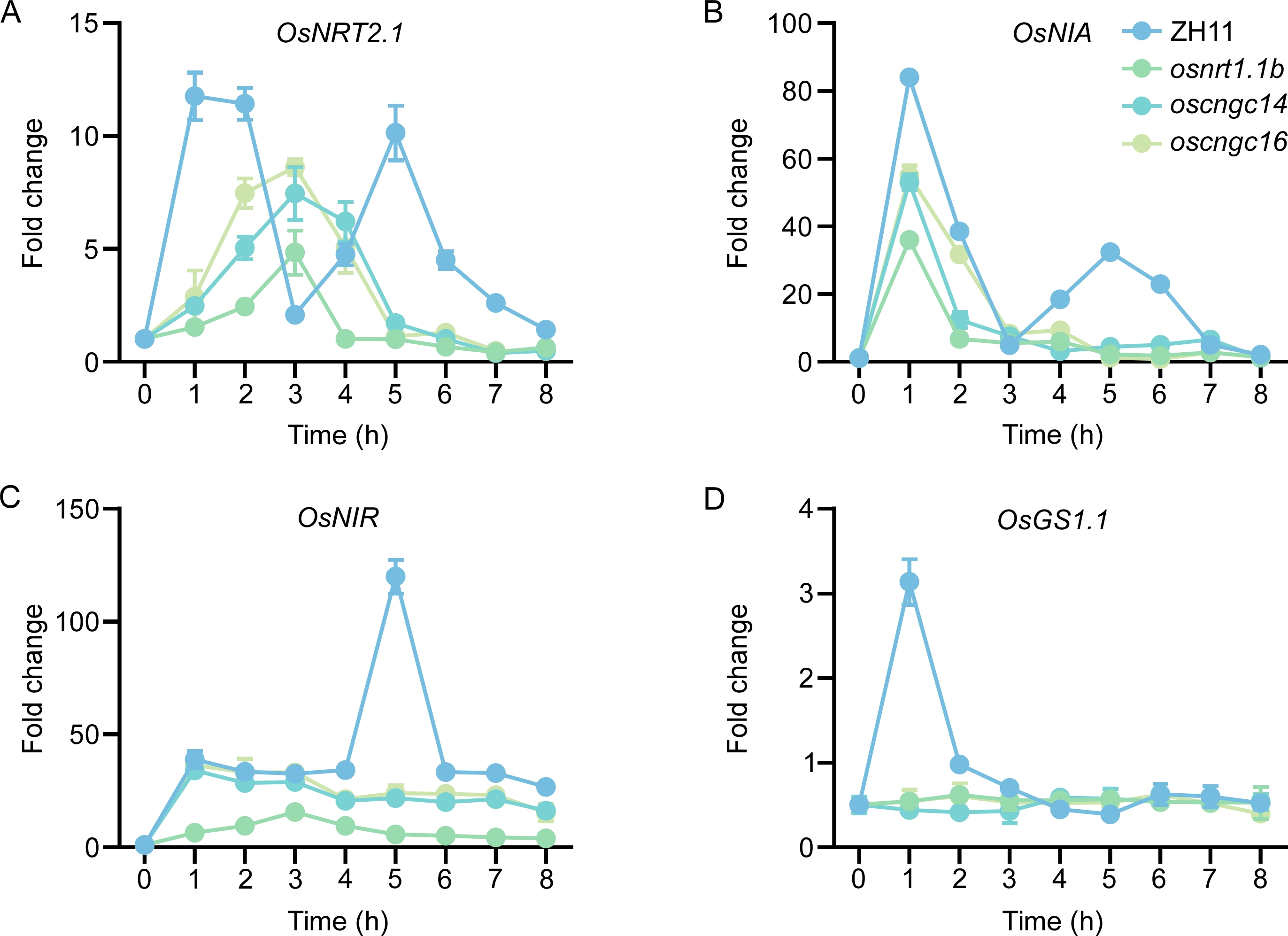


**Supplemental Figure 11. OsNRT1.1B-OsCNGC14/16 mediates the biphasic pattern of nitrogen-responsive genes.**

RT-qPCR analysis of N-related genes in roots of ZH11, *osnrt1.1b*, *oscngc14,* and *oscngc16* plants treated with 5 mM KNO_3_ or KCl (control) for 1-8 hours. Relative expression levels of **(A)** *OsNRT2.1*, **(B)** *OsNIA*, **(C)** *OsNIR*, and **(D)** *OsGS1.1* were normalized to KCl-treated controls (set as 1). Means ± SD. (*n = 3* biological replicates).

**Supplementary Table 1. Primers used for quantitative RT-PCR (RT-qPCR)**

| Gene | Primer sequence |
| --- | --- |
| *OsCNGC1* | 5’ AGTAATGCAGCTAGAAATAAC 3’  5’ CAAAACTAGACCTGATGTCGA 3’ |
| *OsCNGC6* | 5’ TTCTGCGCACAAAGCTCAAT 3’  5’ GCTAAACTTCAGGGTGCTCCT 3’ |
| *OsCNGC7* | 5’ TCTTCCTCTACCTCACGGGG 3’  5’ GGTACCTCCTGGCGATCTTG 3’ |
| *OsCNGC10* | 5’ GTTTCTGCTTCCTCCAGGCT 3’  5’ AGCGAGCAAGAACTCGTCAA 3’ |
| *OsCNGC14* | 5’ TACAAGGCAAGGACGACGAC 3’  5’ CACTCTCGGTCCCAATCCAC 3’ |
| *OsCNGC16* | 5’ GCACTTCCGGTACACCTTCA 3’  5’ AGAAGTCGTCGTCCTGGTTG 3’ |
| *OsNRT1.1B* | 5’ GGCAGGCTCGACTACTTCTA 3’  5’ AGGCGCTTCTCCTTGTAGAC 3’ |
| *OsNRT2.1* | 5’ CACGGGGCTGGAGTACATG 3’  5’ GTTGGGCGGGAGGAACATG 3’ |
| *OsNIA1* | 5’ CCTGGAGAAGATGGGCTAT 3’  5’ GCACAACCATCCATCAATC 3’ |
| *OsNIR1* | 5’ CGAGGAGTAGGAACACAG 3’  5’ TGTCGTCTACTTTACAAGGA 3’ |
| *OsGS1.1* | 5’ CACCAACAAGAGGCACAATG 3’  5’ ACTCCCACTGTCCTGGCAT 3’ |
| *OsNLP1* | 5’ AAGCCGCCACTCTGATGAAA 3’  5’ CTGGGTCTTGCTGTAGGTGG 3’ |
| *OsNLP3* | 5’ GGTGAGGTTGATAAAGTCTGC 3’  5’ AACTCCTTGTCCCTTCTGTAG 3’ |
| *OsNLP4* | 5’ GCATACGATTACCAATTCTGAAGGA 3’  5’ CAAGCAGTTGGAGGGGGTAG 3’ |
| *Actin* | 5’ ACGCCTAAGCCTGCTGGTT 3’  5’ CGCAGCTTCCATTCCTATGAA 3’ |
